# Wild-type p53 oligomerizes more efficiently than p53 hot-spot mutants and overcomes mutant p53 gain-of-function via a “dominant-positive” mechanism

**DOI:** 10.18632/oncotarget.25944

**Published:** 2018-08-10

**Authors:** Dawid Walerych, Magdalena Pruszko, Lukasz Zyla, Michalina Wezyk, Katarzyna Gaweda-Walerych, Alicja Zylicz

**Affiliations:** ^1^ Mossakowski Medical Research Centre, Polish Academy of Sciences, 02-106 Warsaw, Poland; ^2^ International Institute of Molecular and Cell Biology in Warsaw, 02-109 Warsaw, Poland

**Keywords:** p53, oligomerization, FRET, dominant-negative, gain-of-function

## Abstract

Human p53 protein acts as a transcription factor predominantly in a tetrameric form. Single residue changes, caused by hot-spot mutations of the *TP53* gene in human cancer, transform wild-type (wt) p53 tumor suppressor proteins into potent oncoproteins - with gain-of-function, tumor-promoting activity. Oligomerization of p53 allows for a direct interplay between wt and mutant p53 proteins if both are present in the same cells – where a mutant p53's dominant-negative effect known to inactivate wt p53, co-exists with an opposite mechanism – a “dominant-positive” suppression of the mutant p53's gain-of-function activity by wt p53. In this study we determine the oligomerization efficiency of wt and mutant p53 in living cells using FRET-based assays and describe wt p53 to be more efficient than mutant p53 in entering p53 oligomers. The biased p53 oligomerization helps to interpret earlier reports of a low efficiency of the wt p53 inactivation via the dominant-negative effect, while it also implies that the “dominant-positive” effect may be more pronounced. Indeed, we show that at similar wt:mutant p53 concentrations in cells – the mutant p53 gain-of-function stimulation of gene transcription and cell migration is more efficiently inhibited than the wt p53's tumor-suppressive transactivation and suppression of cell migration. These results suggest that the frequent mutant p53 accumulation in human tumor cells does not only directly strengthen its gain-of-function activity, but also protects the oncogenic p53 mutants from the functional dominance of wt p53.

## INTRODUCTION

*TP53* gene, encoding p53 protein, is the most frequently mutated locus overall in human neoplasias [1, 2]. The majority of the *TP53* mutations result in single residue changes in p53 proteins, most of which – including the common “hot-spot” mutations – inactivate DNA-binding and tumor suppressor functions of wild-type (wt) p53, endowing mutant p53 proteins with transforming, gain-of-function (GOF) oncogenic properties [3–5]. Numerous studies - structural and functional - have demonstrated that a tetrameric form of wt p53 is optimal for its effective binding to a target promoter DNA and its function as the tumor-suppressive transcription factor [6–9]. Oligomerization of wt p53 also represents one of its functional weaknesses, as hot-spot mutant p53 variants were observed to inactivate wt p53 by hetero-oligomerization via a dominant-negative (DN) mechanism [10–13]. Chan et al. measured the functional efficiency of the dominant-negative effect to be surprisingly low – as excess of mutant p53 protein was required to inactivate the wt p53 activity [14]. This effect can be partially attributed to a co-translational dimerization of p53, discovered earlier using an *in vitro* translation system [11], which implies that usually mutant p53 enters p53 tetramers as a homodimer – resulting in a limited inactivation of the DNA binding by wt p53, which could be partially retained in the wt p53 homo-dimer [7, 9]. The exchange of monomers within a p53 dimer was indeed found to be ultra-slow in purified p53 proteins *in vitro* [15, 16], and p53 monomer and hetero-dimer concentrations were found to be limited in MCF7 cells - using fluorescence correlation spectroscopy [17] and protein-fragment complementation assay [18]. However, in the mentioned study by Chan and co-workers, only the tumor-derived hot-spot mutants had low efficiency of the functional dominant-negative effect, while wt p53 construct with the deleted transactivation domain (del90) strongly inactivated wt p53 transcriptional activity via hetero-oligomerization [14]. This implied that additional mechanisms may be involved in limiting the dominant-negative effect of the p53 mutants.

It has not been addressed until now whether in a cellular environment p53 oligomerization occurs at the same efficiency for wt-wt, mutant-wt and mutant-mutant combinations – where a bias could contribute to shifting of the functional equilibrium between competing wt and mutant p53 downstream effects. The dominant-negative effect of p53 mutants has also not been directly compared to the efficiency of a “positive-dominance” by wt p53 – an inactivation of the mutant p53 gain-of-function via oligomerization. Mutant p53 has been suggested to be partially inhibited by hetero-oligomerization with wt p53 [19] and several groups have shown that the presence of the expressed wt *TP53* allele is limiting the tumor occurrence driven by p53 mutants in cancer models *in vivo* [20–23].

The FRET (Forster Resonance Energy Transfer) methodology is used to measure intra- and intermolecular interactions *ex vivo* and in living cells [24, 25]. Thanks to dependence of the efficiency of the resonance energy transfer on a number and a spatial positioning of energy acceptors and donors in complexes, FRET and its sister method BRET (Bioluminescence Resonance Energy Transfer), have been utilized to assess oligomerization stoichiometry and dynamics of a number of proteins [26, 27]. Among them were: multimeric transient receptor potential channels (TRPC) family of proteins [28], muscarinic acetylcholine receptors [29], β2-adrenoceptors (β2AR) [30] and other G protein–coupled receptors (GPCRs) [31]. In studies of the p53 protein properties, FRET has been so far used in purified p53 *in vitro*: to assess domain flexibility and determine the conformation states of full-length p53 [32], to detect p53 post-translational modifications [33] and to determine homo-oligomerization dynamics of the isolated p53 tetramerization domain [34]. No FRET studies have been carried out to validate these findings in a living model, measure the wt-mutant p53 hetero-oligomerization and link the observations to functional effects wt and mutant p53.

In this study we describe a FRET-based assay allowing to monitor the efficiency of oligomerization in living cells of p53 proteins fused with CFP and YFP fluorescent protein tags. The method allows to measure dimer- or tetramer-specific p53 oligomerization in a suspension of living cells by a spectrofluorimetric sensitized emission method or in subcellular compartments by confocal microscopy with an acceptor photobleaching method. Both approaches indicate that p53 proteins with wild-type DNA-binding domain oligomerize in cells significantly more efficiently than five tested p53 hot-spot mutant variants. This observation has important functional implications, as at equimolar proportions wt p53 more efficiently suppresses mutant p53 gain-of-function, than mutant p53 inactivates the wt p53 tumor suppressor activity.

## RESULTS

### FRET occurs specifically in tetrameric or dimeric p53 variants, C-terminally tagged with CFP and YFP fluorophores, co-expressed in living cells

To investigate efficiency of the p53 oligomerization in living cells we took advantage of the classic FRET donor-acceptor pair of fluorophores – ECFP and EYFP (named CFP and YFP for clarity in the manuscript) [24, 25]. The main experimental model was H1299 non-small cell lung carcinoma cell line with no endogenous p53, which could be efficiently transfected ectopically with different sets of plasmid constructs. In H1299 cells stability of wt and hot-spot mutant p53 variants is more similar compared to endogenous wt and p53 mutants in cancer cells ([35] and Supplementary Figure 2E) which helps to maintain similar levels of overexpressed wt and mutant p53 variants – an important feature of a model used to compare quantitative effects of p53 variants [14, 36]. Initial experiments were performed using spectrofluorimetry-based sensitized emission method, where CFP (FRET donor) and YFP (FRET acceptor) spectra were measured in suspension of cells in PBS, then CFP spectra (excitation at 425 nm) were corrected for crosstalk, normalized, and the “FRET signal” value was calculated at the YFP acceptor emission peak wavelength of 527 nm (the procedure is shown step-by-step in Supplementary Figure 1A).

**Figure 1:**
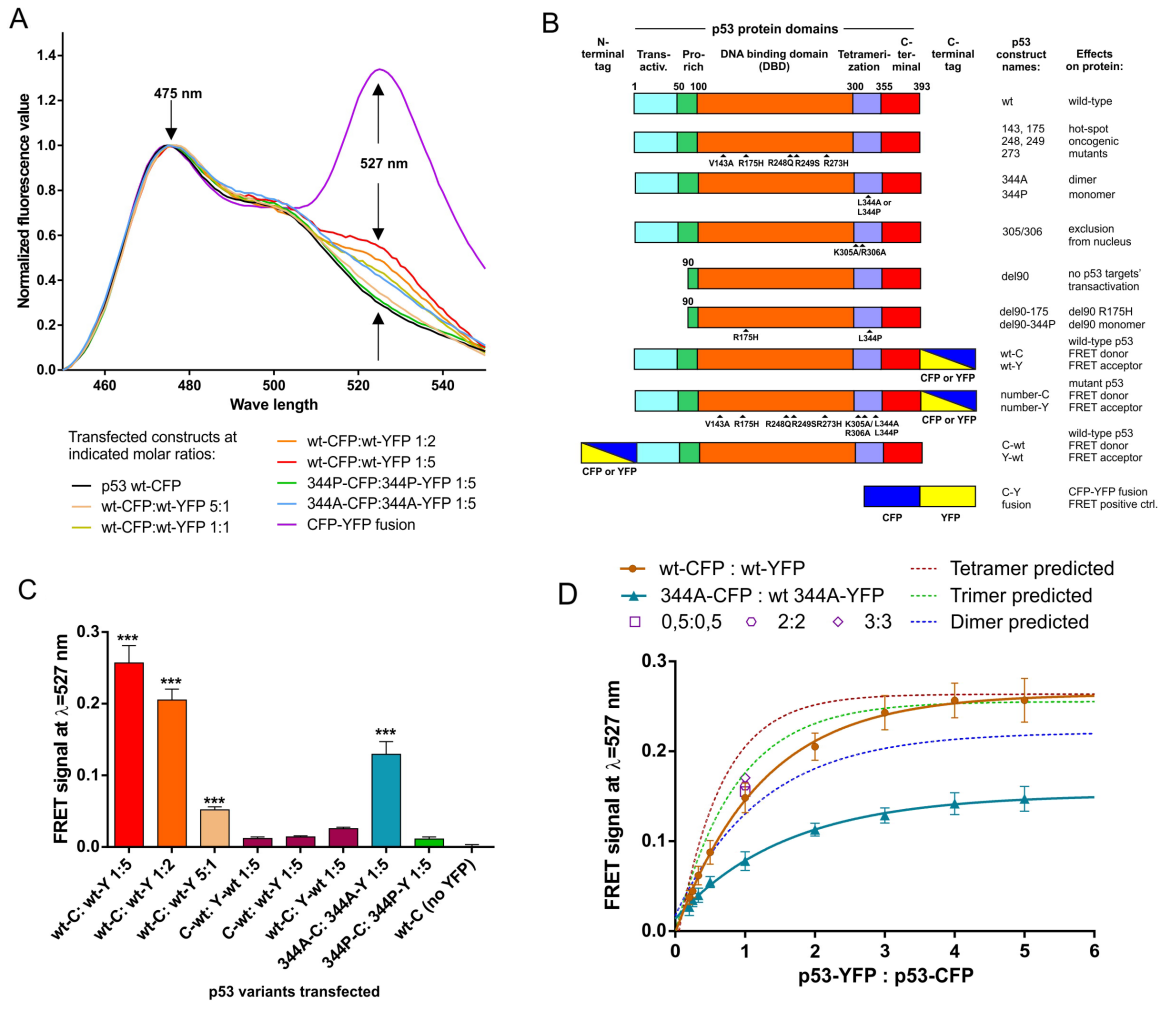
Oligomer-specific FRET signal is detected in living H1299 cells overexpressing C-terminally CFP/YFP tagged p53 proteins, using sensitized emission FRET measurements **(A)** CFP (FRET donor) spectra from H1299 cell suspensions in PBS at RT, transfected with indicated vectors (total of 3 μg transfected vector per 12-well plate well at indicated CFP:YFP construct proportions), were acquired using spectrofluorimetry (Materials and Methods), corrected for crosstalk and normalized (Supplementary Figure 1A). The “FRET signal” value was determined at the YFP (FRET acceptor) emission peak wavelength of λ=527 nm. **(B)** Schematic maps and short descriptions of p53 and control proteins overexpressed ectopically from plasmid constructs used in this study. **(C)** FRET signal values, each with the subtracted average p53 wt-CFP value (CFP background), were compared. Vector transfections at indicated proportions were performed in a biological triplicate. Averages of these 3 results are shown with SD, statistical significance calculated with one-way ANOVA, Bonferroni post-test, ^***^ indicates corrected p-value<0.001 for comparison with wt-CFP, no stars indicates result not being significant in the used test. **(D)** Experimental spectrofluorimetric measurements of the FRET signal in biological triplicate are shown as averages (solid points with SD) for indicated molar proportions of YFP/CFP-tagged wt p53 (tetrameric) or 344A p53 (dimeric) constructs overexpressed in H1299 cells – in the range of 1:5 to 5:1 of YFP:CFP constructs (total of 3 μg transfected vector per 12-well plate well). One-phase association curves were fitted to the experimental results (solid lines) and theoretical oligomerization curves for dimer, trimer and tetramer (dashed lines). The theoretical curves were calculated using the maximum value obtained in the experimental measurements for the p53 wt tetramer, using equation shown in Materials and Methods. Additionally, wt-CFP and wt-YFP were transfected in the equimolar proportions with increasing amount of both vectors (0.5:0.5, 2:2, 3:3 – open symbols). The results were not significantly different from the 1:1 proportion (solid circle) indicating no FRET signal increase due to random CFP:YFP overcrowding in the cells.

Experiments using C-terminally tagged p53 revealed significant FRET signal in wt (tetramerization-capable) p53, increasing with the higher transfected acceptor:donor (YFP:CFP) ratio (Figure 1A), expected from earlier FRET studies on oligomerizing proteins [26, 27]. The FRET signal was also detectable for L344A (tetramerization incapable, dimerization capable) p53 variant [7, 17], albeit weaker than in the case of wt p53 at the same acceptor:donor vector molar ratio (Figure 1A). Monomeric p53 variant L344P [17, 37] did not produce an increased fluorescence signal at 527nm beyond the level of the CFP spectrum of the wt p53-CFP construct transfected alone (Figure 1A). A CFP-YFP fusion construct was used as a high-efficiency FRET positive control (Figure 1A).

Protein domain maps, names and brief descriptions for the constructs used in the study are provided in Figure 1B, while sub-cellular localization in H1299 cells of the most important transfected fluorophore-tagged p53 variants are provided in Supplementary Figure 1B.

To show averages of biological replicates of the transfection experiments as bars with standard deviations, average crosstalk corrected fluorescence value at YFP emission peak 527 nm for the wt p53-CFP variant were subtracted from the crosstalk-corrected values at 527 nm obtained of the consecutive transfections, to obtain “FRET signal” in graph bars – the values of the FRET-derived fluorescence at 527nm. Figure 1C shows a bar-graph representation of biological triplicate of wt:wt, 344A:344A and 344P:344P measurements, whose single replicates were included in Figure 1A. Additionally, low FRET signal was detected for N-terminally tagged wt p53, clearly showing that significant FRET signals could only be obtained between C-terminally tagged p53s, and not in N-N or C-N configurations (Figure 1C). This result is consistent with the closer proximity of the p53 oligomerization domain to the C-terminus of the protein (Figure 1B).

Based on previous studies on oligomeric protein stoichiometry [28, 30, 38], cells were transfected with different molar ratios of vectors with CFP- and YFP-tagged wt p53 (expected tetrameric) or 344A p53 (expected dimeric). The resulting FRET-saturation curves were compared with theoretical models of FRET efficiency at different acceptor:donor ratios for randomly assembling, fully symmetric fluorophore dimerization, trimerization and tetramerization [30]. Neither wt p53 nor 344A p53 non-linear regression one-phase association fit curves matched closely the tetrameric and dimeric theoretical model curves (Figure 1D). Despite that, wt p53 had significantly higher measured and predicted maximum FRET signal values (predicted plateau at 0.2638) than 344A p53 (predicted plateau at 0.1521) at the increasing acceptor:donor ratios, which indicates a higher order oligomerization in wt p53. This also indicated that hetero-oligomerization in the predominantly dimeric 344A p53 is possible in the living cells, but limited below the efficiency of the predicted dimerization model. As suggested by studies on the membrane protein stoichiometry by the FRET methodology [25, 28], we performed additional control of transfecting increasing wt p53-CFP/YFP constructs at equimolar concentrations (0.5:0.5, 1:1, 2:2, 3:3), which all produced FRET values of statistically insignificant difference (Figure 1C). This means that increasing the total p53 protein load did not lead to the increase of FRET signals due to random donor:acceptor aggregation or protein overcrowding in the cells [25, 28].

All these results and used controls indicated clearly that FRET detected in the H1299 living cells between C-terminally CFP/YFP tagged p53 in wt and 344A variants was occurring specifically in the p53 oligomers, whose formation depends on the p53 protein's oligomerization domain.

### Wt p53 oligomerizes more efficiently than p53 hot-spot mutant variants in the living cells

To understand whether p53 oligomerization differs in wt and hot-spot mutant variants frequent in human tumors (V143A, R175H, R248Q, R249S, R273H) [39] we performed FRET signal measurements in H1299 cells transfected with a set of vectors carrying wt and/or mutant p53 variants. We used 1:2 donor:acceptor ratio as a default for these tests, as this allowed for a higher FRET signal value dynamic range than in the 1:1 ratio (Figure 1D), while the donor protein and signal level were high enough for additional co-transfections with other vectors.

**Figure 2:**
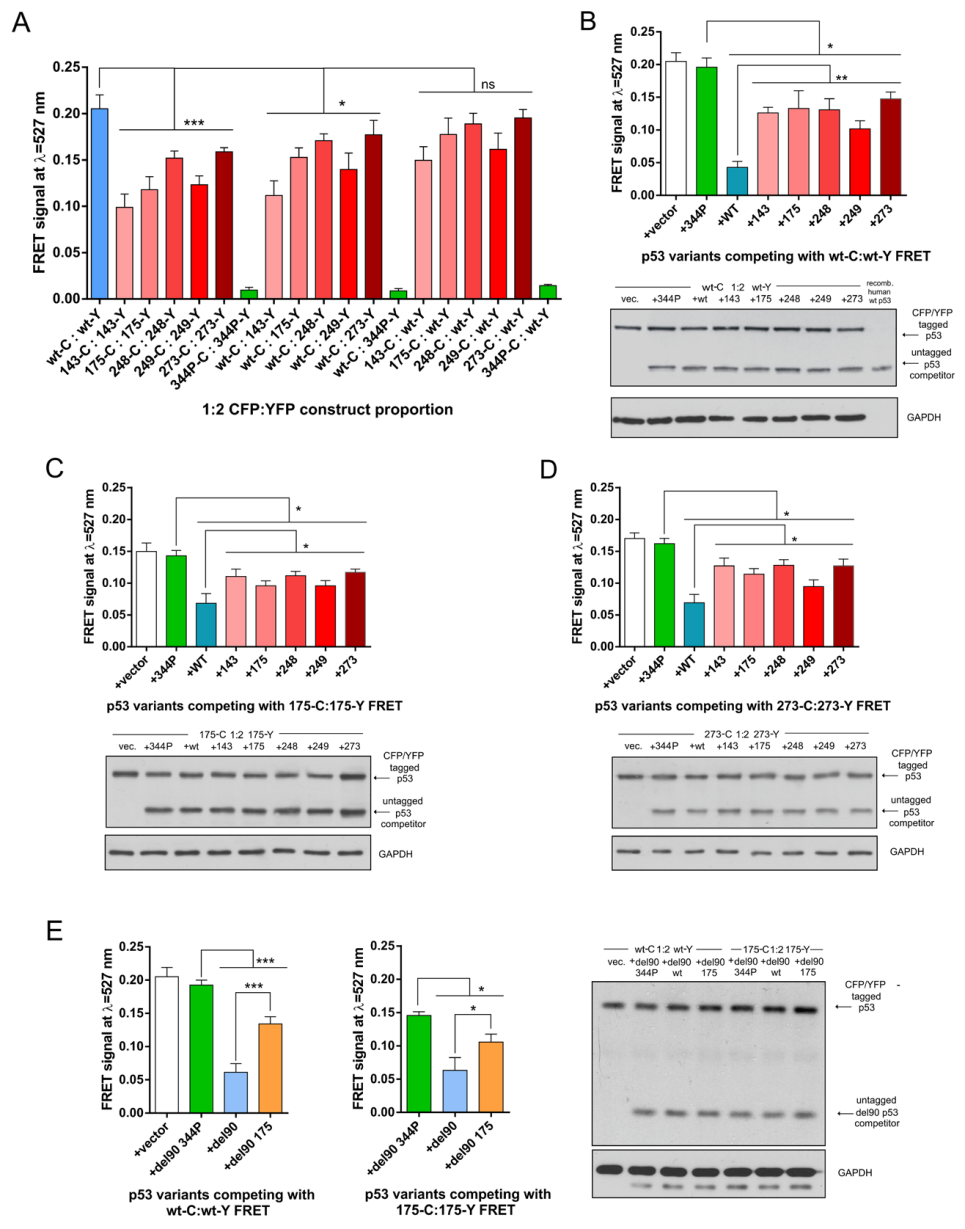
Wild-type oligomerizes more efficiently than p53 hot-spot mutant variants in living cells **(A)** FRET signal values collected from triplicate H1299 cells transfections of indicated vector constructs at the 1:2 CFP to YFP molar vector proportion (1 μg CFP construct : 2 μg YFP construct). Averages of 3 results are shown with SD, statistical significance was calculated with one-way ANOVA, Bonferroni post-test, ^***^ p-value<0.001, ^*^ p-value<0.05, ns - result not significant. **(B)** The FRET competition assay in H1299 cells with p53 wt:wt (transfected 0.5 μg wt-C : 1 μg wt-Y) oligomers using indicated series of untagged constructs or empty vector (1.5 μg co-transfected with FRET constructs). Averages of 3 biological replicates are shown with SD, statistical significance was calculated with one-way ANOVA, Bonferroni post-test, ^**^ p-value<0.01, ^*^ p-value<0.05. Lower panel: western blot analysis for p53 (DO-1 antibody) and GAPDH (loading control) of a total protein lysate from a representative replicate of the FRET measurements shown in the graph and a purified, untagged p53 (positive control). The levels of CFP/YFP tagged and untagged p53 protein variants are similar between all the competition experiments. **(C)** The FRET competition assay in H1299 cells with mutant p53 R175H:R175H (0.5 μg wt-C : 1 μg wt-Y) oligomers using indicated series of untagged constructs or empty vector (1.5 μg co-transfected with FRET constructs). Averages of 3 biological replicates are shown with SD, statistical significance was calculated with one-way ANOVA, Bonferroni post-test, ^*^ p-value<0.05. Lower panel: western blot analysis for p53 (DO-1 antibody) and GAPDH (loading control) of a total protein lysate from a representative replicate of the FRET measurement shown in the graph. **(D)** The FRET competition assay in H1299 cells with mutant p53 R273H:R273H (0.5 μg wt-C: 1 μg wt-Y) oligomers using indicated series of untagged constructs or empty vector (1.5 μg co-transfected with FRET constructs). Averages of 3 biological replicates are shown with SD, statistical significance was calculated with one-way ANOVA, Bonferroni post-test, ^*^ p-value<0.05. Lower panel: western blot analysis for p53 (DO-1 antibody) and GAPDH (loading control) of a total protein lysate from a representative replicate of the FRET measurement shown in the graph. **(E)** The FRET competition assay in H1299 cells with p53 wt:wt (left graph) and mutant p53 R175H:R175H (right graph; 0.5 μg wt-C: 1 μg wt-Y) oligomers using indicated untagged del90 constructs or empty vector (1.5 μg co-transfected with FRET constructs). Averages of 3 biological replicates are shown with SD, statistical significance was calculated with one-way ANOVA, Bonferroni post-test, ^***^ p-value<0.001, ^*^ p-value<0.05. Right panel: western blot analysis for p53 (DO-12 antibody) and GAPDH (loading control) of a total protein lysate from a representative replicate of the FRET measurements shown in the graphs. Visible bands below the main GAPDH bands are caused by binding of an anti-mouse secondary antibody to the remaining DO-12 antibody used earlier to detect del90 p53 on the same membrane.

In homo-oligomerization experiments wt p53 had significantly higher FRET signal than any of the mutant variants (Figure 2A). Wt:mutant hetero-oligomerization experimental setups had significantly lower FRET signal than wt:wt when the p53 mutants were present as tagged with YFP (FRET acceptor) and hence were overexpressed from vectors transfected at 2:1 molar excess over wt p53 (Figure 2A). This suggested that wt p53 could homo-oligomerize with higher efficiency than hetero-oligomerize with mutant p53, which is in turn more efficient than mutant-mutant homo-oligomerization. However, in this type of experiments the difference between FRET signals in wt and mutant p53 proteins could be caused not only by differences in protein levels, localization, activity, subcellular movement or oligomerization rate but also by known structural differences between wt and p53 mutants [40–42]. These protein distortions could affect the FRET efficiency by changing distance or spatial positioning of the FRET donors and acceptors attached to the overexpressed p53 variants [25]. To avoid this problem we used a FRET-competition assay for further experiments – the common method in FRET and BRET studies [27, 28] which allows to measure a decrease of the energy transfer in oligomers caused by competing unlabelled variants.

Wt p53 competed significantly more strongly than any of the five hot-spot mutant p53 variants transfected at equimolar vector amounts with the FRET generated by 1:2 vector proportion of wt-CFP:wt-YFP (Figure 2B). In these experiments western blots were used to confirm uniform levels of the competing p53 variants (Figure 2B–2E). Interestingly, wt p53 variant competed more efficiently than p53 mutants not only with wt:wt FRET but also with FRET generated between mutant variants R175H and R273H (Figure 2C–2D), albeit this effect was less significant than in the case of wt:wt FRET competition assay. The monomeric L344P p53 variant had no effect on the FRET signal and was used as the control reference (Figure 2A–2D).

To exclude that the observed effects are specific only to the H1299 cell background, we performed the FRET-competition assay with wt:wt FRET in the *TP53* -/- *MDM2* -/- mouse embryo fibroblasts – MEFs (Supplementary Figure 2A). This was the only cell type out of several p53-null cell lines tested (others included e.g. Saos-2 and HCT-116 – not shown) which could be efficiently transfected to measure the p53-CFP/YFP high quality spectra, while having comparable levels of the overexpressed wt and mutant p53 variants. In MEFs the overexpressed wt p53 was again competing significantly stronger with wt:wt FRET than the two tested mutant variants – confirming earlier results from H1299 cells (Supplementary Figure 2A).

To make sure that the untagged p53 variants indeed hetero-oligomerize with the fluorophore-tagged p53 variants, a control co-immunoprecipitaion (co-IP) experiments were carried out in H1299 cell lysates using anti-GFP antibodies. Indeed, there was no co-IP of the L344P monomeric variant while other p53 variants co-precipitated with the YFP-tagged p53. Additionally, despite the co-IP method being primarily qualitative rather than quantitative, the untagged wt p53 co-precipitated stronger with C-terminally YFP-tagged wt p53 than R175H or R273H mutant variants (Supplementary Figure 2C). This result was supported by the efficient co-IP of the untagged wt p53 with the N-terminally FLAG-tagged wt p53 in H1299 cell lysates, using anti-FLAG antibodies (Supplementary Figure 2D).

The western blots performed for the FRET-competition assay and for the control co-IP, indicated that the difference in wt and mutant p53 oligomerization efficiency cannot be explained by the different protein levels of overexpressed p53 wt and mutant variants, hence we asked if the increased cytoplasmic localization of at least V143A, R175H and R249S p53 variants, compared to the predominantly nuclear wt p53 (Supplementary Figure 1B), could be decisive for the increased wt p53 oligomerization. However, wt p53 was still significantly more efficient competitor than any of the five p53 mutants with the FRET generated between K305A/R306A p53 variant (Supplementary Figure 2B), excluded from cell nuclei by an inactivated p53 nuclear localization signal (Supplementary Figure 1B) [43]. This result showed that differences in the localization of p53 are not decisive for the higher efficiency of the wt p53 oligomerization.

Since overexpressed wt p53 is inducing tumor suppressive downstream target transcription and tumor-suppressive phenotypic effects in cells [44] (see also Figure 4 and Supplementary Figure 5), we next investigated if this transcription-based activity was responsible for the observed increased wt p53 oligomerization. To this end we used del90 p53 variants, with the deleted entire transactivation domain (Figure 1B), which were shown to be deprived of the wt p53 transactivation properties, but retained ability to enter and functionally inactivate wt p53 tetramers, and were localized to the nuclei of H1299 cells [14]. Del90 p53 was a better competitor with FRET generated by wt:wt and R175H:R175H than del90 p53 with the hot-spot R175H mutation (Figure 2E), indicating that the presence of the N-terminal transactivation domain in p53 and the physiological consequences of its presence are not required by wt p53 to enter p53 oligomers more efficiently than mutant p53.

Altogether, the results of the series of wt p53 and p53 hot-spot mutants oligomerization measurements by the direct FRET and the FRET-competition assay indicate that wt p53 is significantly more efficiently forming p53 oligomers containing at least single CFP-YFP FRET pairs of monomers, than the tested mutant variants.

### Single-cell, acceptor photobleachaing FRET measurements confirm the biased oligomerization of p53 in nuclei of H1299 cells

All the FRET measurements described above in the Results section were performed in cell suspensions, using spectrofluorimetry with the sensitized emission protocol, thus resulting in FRET signal averages from large populations of transfected cells (Figures 1–2). To verify if the observed effects are not caused by a specific subpopulation of cells or local subcellular events, such as protein aggregation, we re-performed the most important FRET measurements using confocal microscopy with the acceptor-photobleaching protocol in single, live, H1299 cells, maintained under a microscope in Lab-Tek chambers.

**Figure 3:**
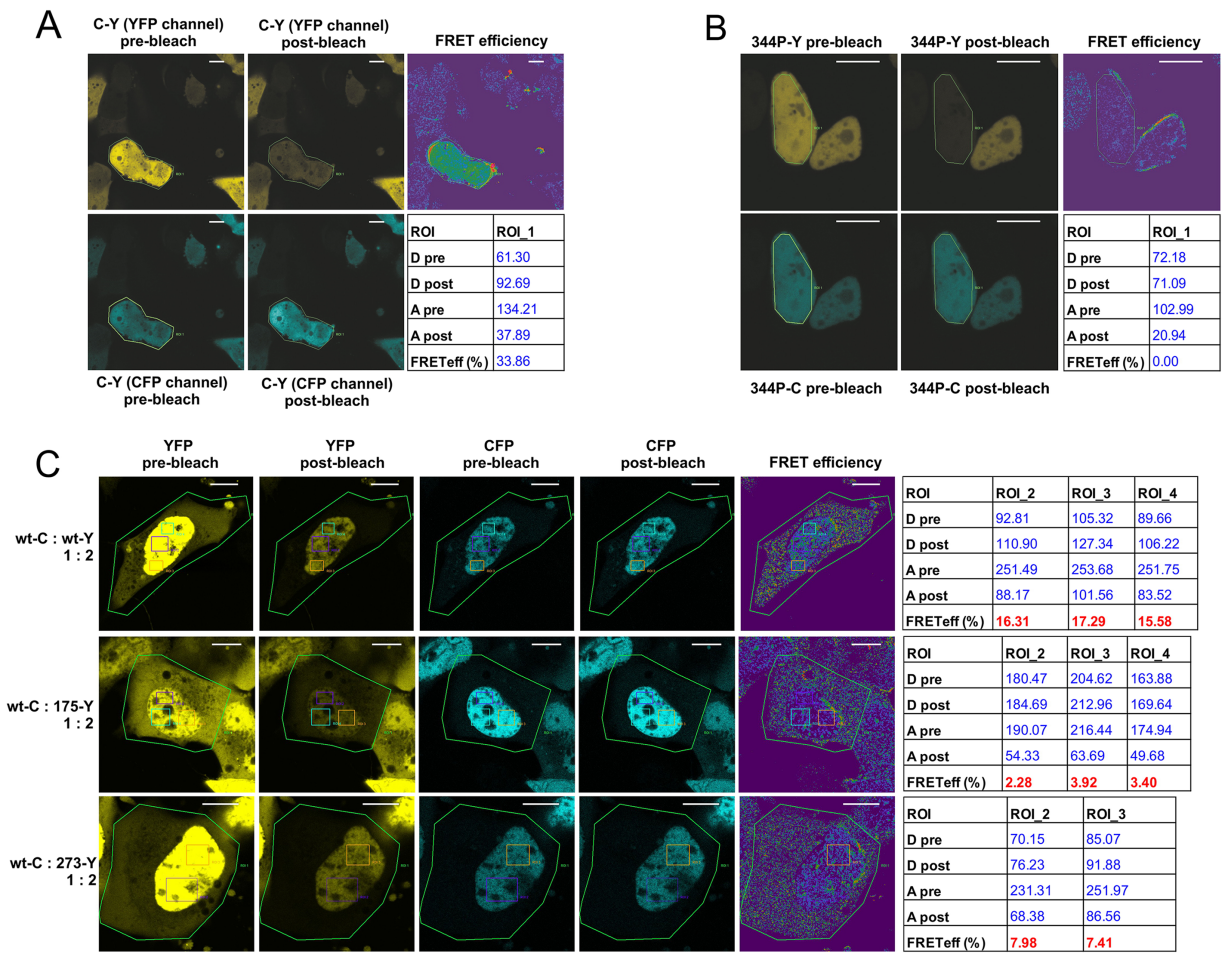

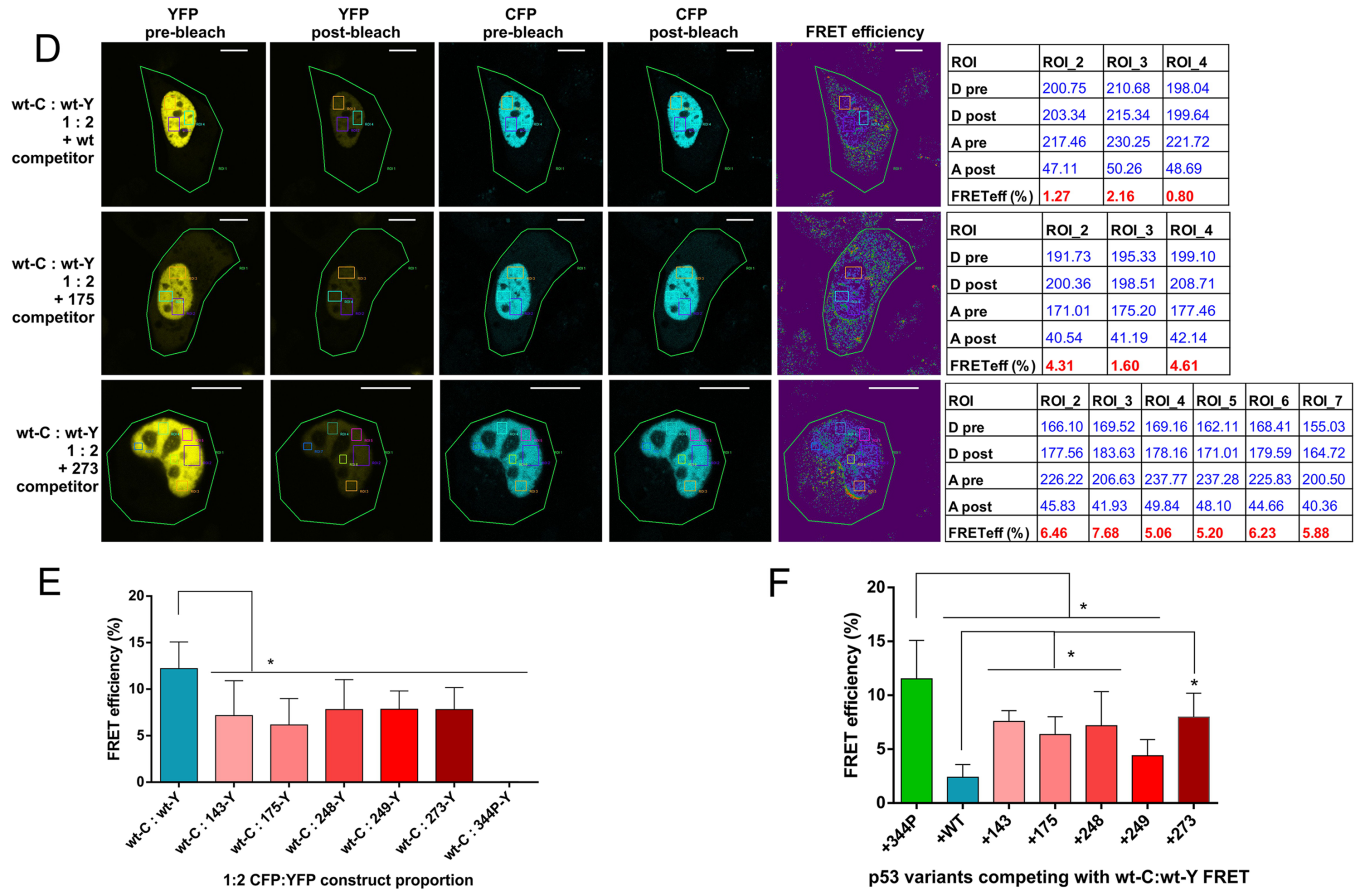
Confocal microscopy acceptor photobleaching protocol shows biased p53 oligomerization in the nuclei of individual, living H1299 cells **(A)** Confocal microscopy photos and calculated results of the acceptor photobleaching FRET efficiency measurement in live H1299 cell growing in a Lab-Tek chamber, transfected with the vector overexpressing CFP-YFP fusion protein (FRET positive control). Pre- and post-bleach photos are shown for the CFP channel (donor; blue) and the YFP channel (acceptor; yellow). FRET efficiency color map and the result table show the FRET efficiency calculated for the photobleached area indicated as ROI (Region of Interest). Bar size - 10 μm. **(B)** Confocal microscopy photos and results of the acceptor photobleaching FRET efficiency measurement in live H1299 cell growing in a Lab-Tek chamber, transfected with vectors overexpressing 344P-C and 344P-Y monomeric p53 variants (vector molar proportion 1:2). Pre- and post-bleach photos are shown for the CFP channel (donor; blue) and the YFP channel (acceptor; yellow). FRET efficiency color map and the result table show the FRET efficiency calculated for the photobleached nucleus area indicated as ROI. Bar size - 10 μm. **(C)** Confocal microscopy photos and results of the acceptor photobleaching FRET efficiency measurement in live H1299 cell growing in a Lab-Tek chamber, transfected with vectors overexpressing p53 wt-C and indicated YFP-tagged p53 variants (vector molar proportion 1:2). Pre- and post-bleach photos are shown for the CFP channel (donor; blue) and the YFP channel (acceptor; yellow). FRET efficiency color map and the result table show the FRET efficiency calculated for the ROIs in the nuclei, after photobleaching of whole cells (ROI 1). Bar size - 10 μm. **(D)** Confocal microscopy photos and results of the acceptor photobleaching FRET efficiency measurement in live H1299 cell growing in a Lab-Tek chamber, transfected with vectors overexpressing p53 wt-C, wt-Y and indicated untagged p53 FRET-competitor variants (vector molar proportion 1:2:3). Pre- and post-bleach photos are shown for the CFP channel (donor; blue) and the YFP channel (acceptor; yellow). FRET efficiency color map and the result table show the FRET efficiency calculated for the ROIs in the nuclei, after photobleaching of whole cells (ROI 1). Bar size - 10 μm. **(E)** Bar graph representation of the mean FRET efficiency results of the wt p53 hetero-oligomerization experiments shown in (C) – including the set of five hot-spot p53 mutant variants and the monomeric variant 344P. The bars are means with SD of ROI results from at least 3 cells each from 2 biological replicates of the experiments. Statistical significance was calculated with one-way ANOVA, Bonferroni post-test, ^*^ p-value<0.05. **(F)** Bar graph representation of the mean FRET efficiency results of the FRET competition assay experiments shown in (D) – including the set of five untagged, competing five hot-spot p53 mutant variants and the monomeric variant 344P. The bars are means with SD of ROI results from at least 3 cells each from 2 biological replicates of the experiments. Statistical significance was calculated with one-way ANOVA, Bonferroni post-test, ^*^ p-value<0.05.

First, as a proof-of-principle, we confirmed the high FRET efficiency in the positive control (CFP-YFP fusion construct) and null FRET efficiency in the negative control (p53 L334P monomeric variant) (Figure 3A–3B). It became apparent that subcellular FRET results using the acceptor photobleaching protocol are affected by small patches of a high measured FRET efficiency, usually appearing in the membrane vicinity or regions of uneven/low fluorophore signal (Supplementary Figure 3A). To avoid these possible artifacts, while whole cells were by default photobleached to the 30% of the initial YFP intensity, the final FRET efficiencies were measured in rectangular regions of interest (ROIs) with uniform donor and acceptor signals (Supplementary Figure 3A). The ROIs were placed in the cell's nuclei – which contained high and relatively uniform fluorophore signal levels in case of all the used p53 variants. Additionally, to diminish variation of the FRET efficiency caused by different proportions of CFP:YFP in individual cells (example shown in Supplementary Figure 3B), data for the final results (Figure 3C–3E) were collected from cells with proportion of the CFP:YFP signals close to an average in a given experiment.

In the first series of experiments we measured FRET efficiencies in p53 wt-CFP:wt-YFP and wt-CFP:mutant-YFP oligomers (Figure 3C). As in the case of the sensitized emission experiments – wt:wt oligomers produced significantly higher FRET efficiencies, shown in detail for wt p53 homo-oligomerization and hetero-oligomerization with two representative mutant p53 examples (Figure 3C), and for all five p53 mutants used in the study in the bar graph with the average results of multiple ROIs from at least 3 cells each from 2 biological replicates of the transfection experiments (Figure 3E).

Next, we performed the FRET-competition assay with the FRET generated in the wt:wt oligomers. The results of this test showed that overexpressed, unlabelled wt p53 was able to compete more efficiently with the FRET derived from wt-CFP:wt-YFP oligomers in H1299 cells nuclei, than the hot-spot mutant variants: R175H and R273H - shown in detail for the representative examples (Figure 3D), and for all five p53 mutants in the bar graph (Figure 3F).

The confocal microscopy experiments confirmed the sensitized emission protocol results and showed that the more efficient oligomerization of wt p53 compared to hot-spot mutant variants in representative transfected H1299 cells was taking place in the cell nuclei, where signals from fluorescently tagged p53 proteins were high and evenly distributed.

### Biased p53 oligomerization affects mutant p53 gain-of-function inactivation by wt p53

The results from the FRET-based p53 oligomerization tests supported by the co-IP results (Figures 2–3), indicated the ability of wt p53 to form oligomers with an increased efficiency compared to hot-spot mutant variants. In the next step we verified how this effect translates to the tumor suppressive function of wt p53 and oncogenic gain-of-function of p53 mutants.

**Figure 4:**
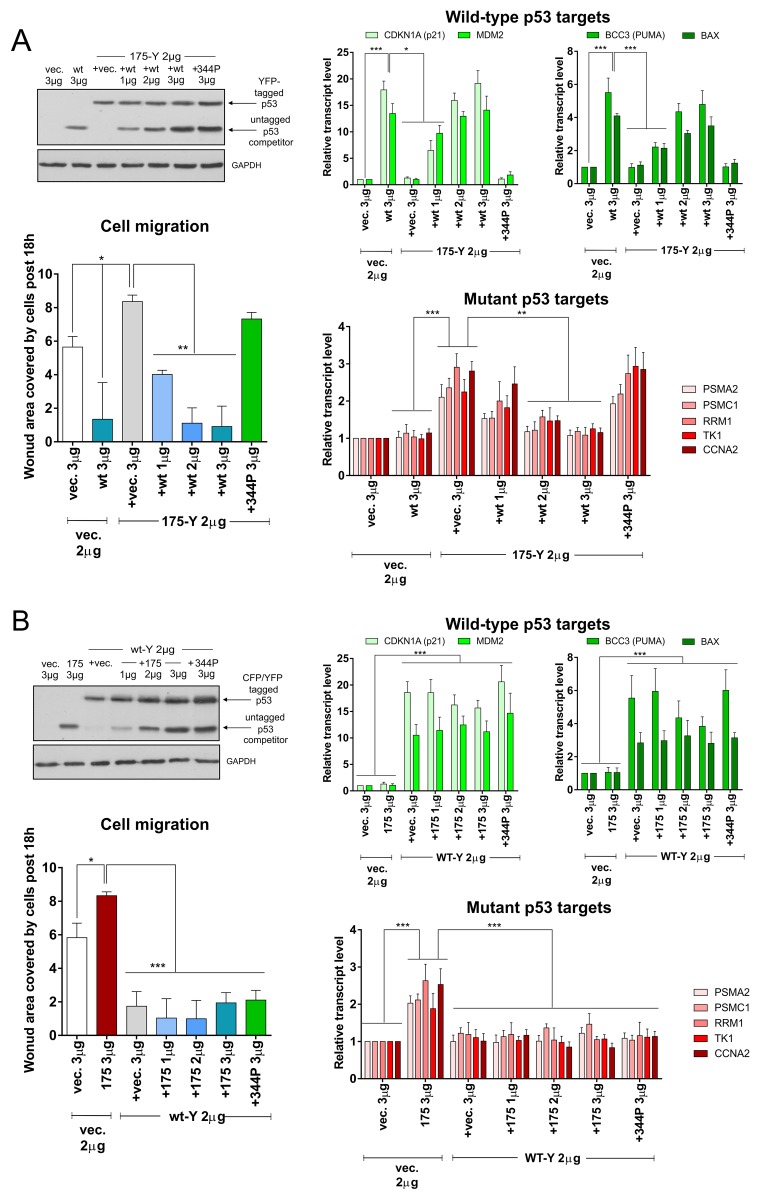
Wild-type and mutant p53 hetero-oligomerization leads to inefficient wt p53 activity inhibition and efficient mutant p53 gain-of-function inhibition **(A)** H1299 cells were transfected with indicated amounts of empty vector (vec.) and/or vectors encoding p53 protein variants – untagged wt or 344P with/without 175-YFP mutant. The western blot shows p53 (DO-1 antibody) and GAPDH (housekeeping control) levels in the representative competition titration experiment. Expression bar graphs show normalized averages with SD of the mRNA levels for wt p53 target genes (strongly induced: *CDKN1A* and *MDM2;* moderately induced: *BCC3* and *BAX*) and mutant p53 target genes (*PSMA2, PSMC1, RRM1, TK1, CCNA2*) in 3 biological replicates of the wt p53 titration vs p53 175-Y mutant variant. The cell migration graph shows average results with SD of area covered by cells post 18h of the wound-healing (scratch) assay in 3 biological replicates of the wt p53 titration vs p53 175-Y mutant variant. Statistical significance was calculated with one-way ANOVA, Bonferroni post-test, ^***^ p-value<0.001, ^**^ p-value<0.01, ^*^ p-value<0.05. The result indicates that significant inhibition of the mutant p53 GOF effects (gene expression and cell migration induction) by wt p53 is present already at equimolar amount of wt *vs.* mutant p53. **(B)** H1299 cells were transfected with indicated amounts of empty vector (vec.) and/or vectors encoding p53 protein variants – untagged 175H or 344P with/without wt-YFP. The western blot shows p53 (DO-1 antibody) and GAPDH (housekeeping control) levels in the representative competition titration experiment. Expression bar graphs show normalized averages with SD of the mRNA levels for wt p53 target genes (strongly induced: *CDKN1A* and *MDM2;* moderately induced: *BCC3* and *BAX*) and mutant p53 target genes (*PSMA2, PSMC1, RRM1, TK1, CCNA2*) in 3 biological replicates of the mutant R175H p53 titration vs wt-Y. The cell migration graph shows average results with SD of area covered by cells post 18h of the wound-healing (scratch) assay in 3 biological replicates of the R175H mutant p53 titration vs wt-Y. Statistical significance was calculated with one-way ANOVA, Bonferroni post-test, ^***^ p-value<0.001, ^*^ p-value<0.05. The result indicates that inhibition of the wt p53 effects (gene expression induction and cell migration inhibition) by mutant p53 is ineffective even at excess of mutant *vs.* wt p53.

We transfected the H1299 cells at analogous vector proportions as in the FRET-competition assays and measured transcriptional activation of *CDKN1A* (p21), *MDM2*, *BAX* and *BCC3* (PUMA) genes by wt p53, against mutant p53 target genes: 26S proteasome subunit genes (*PSMA2* and *PSMC1*) - earlier described by us to be transcriptionally activated by mutant p53 in cooperation with Nrf2 [45, 46], nucleotide biosynthesis genes (*RRM1* and *TK1*) activated by mutant p53 in cooperation with ETS2 [47] and Cyclin A gene (*CCNA2*) activated by mutant p53 in cooperation with NF-Y [48]. Additionally, we tested by a wound-healing (scratch) assay how the different wt:mutant proportions affect the ability of H1299 cells to migrate, a property known to be affected by wt/mutant p53 in various cell lines, including H1299 [4, 49–51]. Besides untagged p53s we used wt and mutant p53 variants tagged with YFP at C-termini which activated the tested genes and affected the wound-healing assay in a similar manner to wt and mutant p53 with no tags (Figure 4A, B and Supplementary Figure 5A).

In the setup with a steady level of the p53 R175H-YFP construct, the increasing level of wt p53, but not monomeric 344P p53, was able to activate wt p53 transcriptional targets and decrease cell migration, despite the presence of mutant p53 (Figure 4A, Supplementary Figure 4A). However, the mutant p53 targets and migration could be induced significantly compared to the empty vector control only if wt p53 was absent or in the presence of the monomeric L344P p53 variant (Figure 4A, Supplementary Figure 4A). These results were additionally confirmed in setups with YFP-tagged R273H p53 mutant variant, untagged p53 proteins (in H1299 cells) and YFP-tagged R175H variant in H358 *TP53*-null non-small cell lung cancer cells (Supplementary Figure 5A-5C and Supplementary Figure 6A-6C). In H358 cells three mutant p53 target genes were significantly induced (*PSMA2*, *PSMC1* and *TK1)*, and hence are shown in Supplementary Figure 5C. In a reversed setup – when the wt-YFP construct was at the steady level – only the excess of R175H p53 over the wt-YFP level could visibly but not significantly inhibit the wt p53 targets’ transactivation (Figure 4B). Mutant p53 targets and cell migration were instead kept at the wt p53 levels even upon introduction of the excess of the R175H p53 mutant variant (Figure 4B, Supplementary Figure 4B).

These results, while being in agreement with earlier notions that a large excess of oligomerization-capable mutant p53 is needed to inactivate the wt p53 activity [14], additionally indicated that proportionally less oligomerization-capable wt p53 is required to inactivate the mutant p53 gain-of-function.

**Figure 5:**
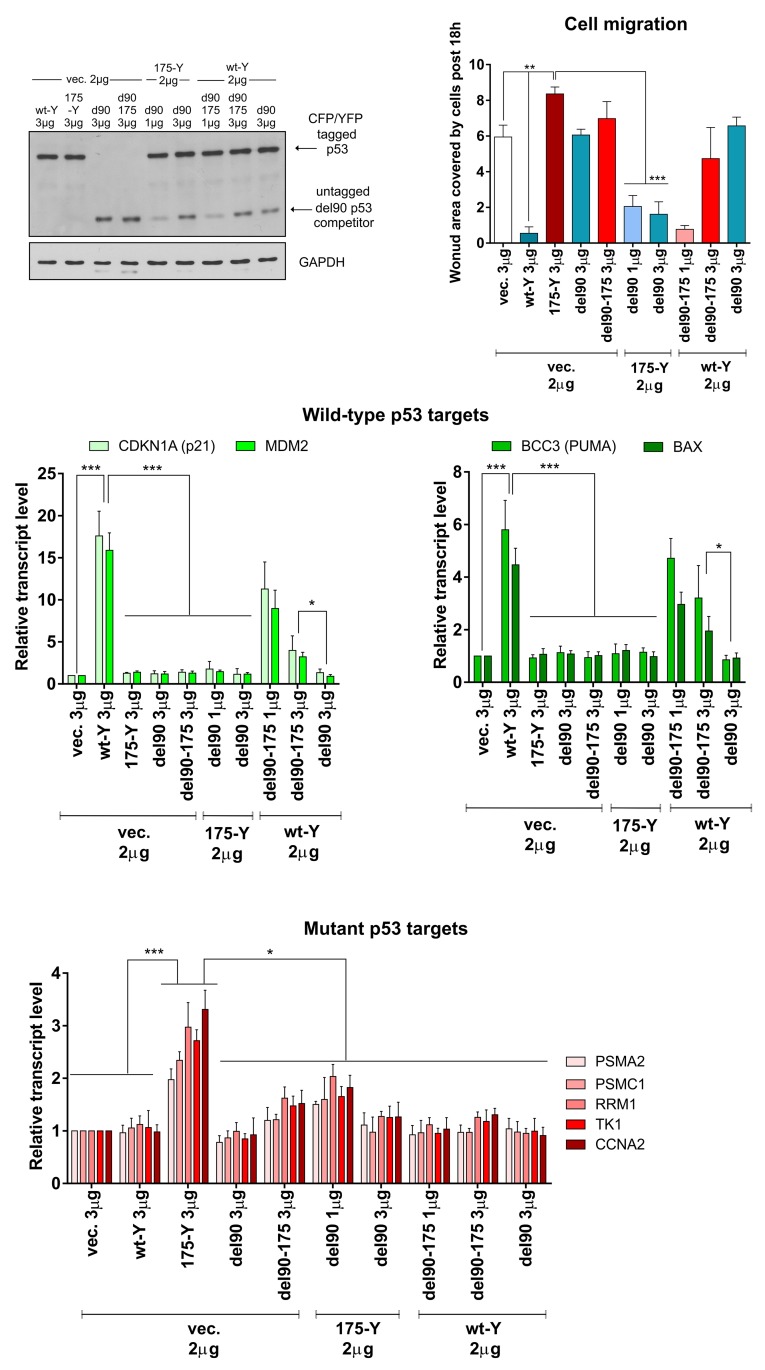
Del90 p53 variant, without the wt p53 transactivation domain and activity, efficiently inactivates the mutant p53 gain-of-function H1299 cells were transfected with indicated amounts of empty vector (vec.) and/or vectors encoding p53 protein variants – untagged del90 or del90 with R175H mutation, with/without 175-YFP or wt-Y. The western blot shows p53 variants (DO-12 antibody) and GAPDH (housekeeping control) levels in the representative experiment. Expression bar graphs show normalized averages with SD of the mRNA levels for wt p53 target genes (strongly induced: *CDKN1A* and *MDM2;* moderately induced: *BCC3* and *BAX*) and mutant p53 target genes (*PSMA2, PSMC1, RRM1, TK1, CCNA2*) in 3 biological replicates. The cell migration graph shows average results with SD of area covered by cells post 18h of the wound-healing (scratch) assay in 3 biological replicates. Statistical significance was calculated with one-way ANOVA, Bonferroni post-test, ^***^ p-value<0.001, ^**^ p-value<0.01, ^*^ p-value<0.05. The result indicates that significant inhibition of the mutant p53 GOF effects (mutant p53-specific gene expression and cell migration induction) is possible by the del90 p53, while the wt p53 effects (wt p53-specific gene expression induction and cell migration inhibition) are more efficiently inhibited by del90 p53 rather than del90 p53 with the hot-spot R175H mutation.

To validate if the target genes’ transactivation by wt p53 is required for the inactivation of the mutant p53 gain-of-function, we used the p53 del90 variants. It turned out that del90 p53 indeed inactivates the R175H p53-dependent transactivation of the proteasome genes and the induction of migration (Figure 5, Supplementary Figure 7). Additionally, del90 p53 inactivated wt p53 more efficiently than del90 R175H (Figure 5). These results indicated that the mutant p53 gain-of-function activity, similarly to the mutant:mutant FRET (Figure 2E), is inactivated by del90 p53 variant with the wt DNA-binding domain, but deprived of the transactivation ability. The results also confirmed functionally the oligomerization assay result (Figure 2E) by showing that del90 p53 with the mutant DNA-binding domain is less efficient in inactivation of the full-length wt p53 than del90 with the wt p53 DNA-binding domain.

Altogether, the results of the functional experiments showed relatively weak inactivation of wt p53 via a dominant-negative mechanism compared to the more efficient inactivation of the mutant p53 gain-of-function by wt p53, in a “dominant-positive” manner.

## DISCUSSION

The FRET-based assay results presented in this study demonstrate a previously unknown ability of wt p53 to oligomerize more efficiently in the living cells compared to hot-spot mutant p53 variants, frequently present in human neoplastic cells [39]. To generate a detectable FRET signal, at least pairs of CFP and YFP fluorophores have to be present within nanometer-scale range of the radiationless energy transfer in populations of proteins [24, 25]. In our experiments the significant FRET could be produced only by the oligomerization-capable p53 variants – tetrameric and dimeric, tagged with fluorophores only at the C-termini, and the maximum detected FRET signal of teterameric p53 was significantly higher than the dimeric p53 in the FRET-saturation experiments. Hence, we concluded that the observed FRET in the H1299 living cells is generated specifically in dimers and tetramers of p53.

The higher observed FRET signal (in the sensitized emission experiments) and FRET efficiency (in the acceptor photobleaching experiments) in wt p53 oligomers can be interpreted either as a higher exchange rate of monomers within oligomers, or as a higher rate of oligomer formation, and as both of these effects combined. The studies on full-length p53 dimerization and tetramerization performed *in vitro* [11, 15, 16] and in the MFC7 cells [17, 18] have suggested that the dimer formation by p53 monomers is largely irreversible and co-translational, and that p53 tetramers are formed mostly by homodimers. In the same time the *in vitro* analyses demonstrated a slow dissociation of dimers into momoners [16], and a fraction of monomeric wt p53 was detectable in live cells [17]. Our results confirm that the p53 hetero-dimerization in the live cells is possible, albeit nearly 1,5x less efficient than in the theoretically predicted hetero-dimerization FRET saturation curve. Hence, the contribution of the hetero-dimerization to the measured oligomerization efficiency is limited below the predicted values, and is the likely reason why the hetero-tetramerization FRET-saturation curve does not fit the predicted values for the tetramer. Thus, our method does not exclude neither higher exchange rate of monomers in wt p53 dimers/tetramers nor more abundant wt p53 dimers/tetramers as the cause of the observed stronger direct FRET or FRET competition by wt p53. This problem could be possibly addressed by the future research on full-length wt and mutant p53 homo- and hetero-tetramerization using single-molecule spectroscopy methodology, which has been recently employed to show structural properties and kinetics of the isolated p53 tetramerization domain [34]. Several studies on oligomerization of proteins have been also carried out using FRET spectrometry in living cells, allowing to understand the spatial configuration and quantitative contribution of the proteins’ oligomeric states [52–54], suggesting possible solutions for further research on the p53 oligomers.

Wt p53 and p53 hot-spot mutant variants compared here for their oligomerization properties differ in a number of ways - including structural and biophysical properties [40–42], different sub-cellular localization and aggregation patterns [55–57], target gene transactivation [4, 58] and different specific interactors [45, 59–61]. Hence, there are various possible mechanistic explanations how oligomerization could be differentially regulated in wt and mutant p53 variants. Here, we excluded several possibilities in the H1299 lung carcinoma cell background used for the majority of the experiments. We showed that protein localization is not the key parameter, as wt p53 competed more efficiently compared to hot-spot p53 mutants with the FRET generated by wt p53 (mostly nuclear) or the predominantly cytoplasmic p53 variant K305A/R306A. Additionally, not only “conformation” mutant p53 variants used (V143A, R175H and R249S) with unfolded DNA-binding domain [40, 41] which localized to the nucleus and the cytoplasm of the H1299 cells, but also the nearly native, “contact” p53 mutants (R248Q and R273H) [40, 41] which localized similarly to wt p53 mostly to the nucleus, were producing weaker FRET signals in homo-oligomers and were competing with lower efficiency with the FRET derived from any p53 oligomers. By performing experiments with del90 p53 variants without the p53 transactivation domain we also excluded that the wt p53 tumor-suppressive transactivation, which leads to severe physiologic consequences in cells - apoptosis, cell cycle arrest and metabolic reprogramming [44] and thus possibly influences the p53-CFP/YFP turnover, localization or folding - is decisive for the more efficient wt p53 oligomerization. Therefore, the most likely mechanistic reason for the biased p53 oligomerization is the composition of the DNA-binding domain and its consequences – at the level of protein structure and interaction with other molecules. It has been shown that not only the “conformational” p53 mutants, such as R175H, possess specific protein interactors [62], but it is also the case in the p53 mutant variant with the nearly native DNA-binding domain - R273H [59, 61]. Both of these types of p53 mutants were also shown to possess numerous specific and common gain-of-function transcriptional targets via an indirect DNA binding, distinct from wt p53 [4, 45]. Given that interacting factors and resulting post-translational modifications may affect the oligomerization state of p53 [63–65], it is likely that the distinct interactomes of wt and hot-spot mutant p53s hold the key to the differences in their oligomerization efficiencies.

As shown in the functional part of this study – the difference in oligomerization properties of wt and mutant p53 may help to understand the equilibrium of wt- *vs.* mutant p53-dependent processes of tumor suppression *vs.* oncogenic negative-dominance and gain-of-function. The analyses of Li-Fraumeni patients with inherited *TP53* mutations as well as mouse Li-Fraumeni models consequently demonstrate that the increased tumor development in Li Fraumeni syndrome involves selective pressure to eliminate the activity of the wt *TP53* allele from the heterozygous setup with mutant *TP53*. It is achieved via a loss-of-heterozygosity (LOH) and other mechanisms of wt p53 inactivation in Li-Fraumeni patients [66, 67] and mouse models [21]. In mice with the *Trp53* mut/wt genotype onset of tumors was found to be later and survival of the animals was longer, compared to the mut/- genotype, while the loss of the wt allele or accumulation of the mutant p53 protein level occurred in the majority of tumors with the mut/wt genotype [20, 21, 68]. Accumulating data from mouse models, in concordance with human patient data, point to LOH as a functional prerequisite to the mutant p53 gain-of-function [69–71]. Furthermore, a hypomorphic wt *Trp53* allele, expressed at low level compared to a mutant allele, was able to suppress tumor growth, but did not lead to tumor regression in mice with a knock-in *TP53* missense mutant [22]. A recent research by Turrell et al., using Ras and mutant p53-driven lung carcinoma mouse model, extended these findings with a stronger induction of the wt p53 expression in the presence of mutant p53 – demonstrating that wt p53, though still at lower level than mutant p53, largely avoids and suppresses the effects of mutant p53 on transcription and tumor growth [23]. The authors hypothesized that wt-mutant p53 hetero-tetramers may partially retain the wt transcriptional activity and/or the p53 oligomerization may be biased [23]. Our research for the first time shows that indeed the p53 oligomerization is biased, at least in lung carcinoma H1299 and mouse embryo fibroblasts backgrounds, and this effect is reflected by the wt and mutant p53 activities in lung cancer cells. We show that p53 hot-spot missense mutants in full-length and del90 p53 proteins are relatively ineffective in inhibition of the wt p53 transcriptional and migration-suppressing activities, which largely confirms earlier mechanistic findings [14] and a low mutant p53 dominant-negative oncogenic impact in human tumors or cell lines [72, 73]. However, we also demonstrate that the oligomerization-capable p53 proteins with the wt DNA-binding domain – full length or del90 – are efficient in suppressing mutant p53-mediated gene expression and cell migration, which represent some of the mutant p53's gain-of-function hallmarks [45]. This reversed negative-dominance we refer to as the “dominant-positive” effect of wt p53.

Altogether, our results demonstrate that to inactivate mutant p53, wt p53 has to be capable of oligomerization via its tetramerization domain, but does not have to be capable of the direct transcriptional activation. Since our study defines the wt p53 oligomerization as more efficient, we show that equimolar level of wt or del90 p53 is enough to inactivate the mutant p53 gain-of-function, while even the excess of mutant p53 is not able to effectively inactivate wt p53. This is consistent with the fact that hot-spot p53 mutants accumulate in various tumor types in mouse models [20, 68] and human patients [74–79], while there is a selective pressure to eliminate the wt *TP53* allele in transformed cells [69–71]. Our research implies that these mechanisms do not only allow boosting the mutant p53 gain-of-function [80], but also prevent efficient inactivation of mutant p53 gain-of-function by wt p53 during cell transformation. This suggests that strategies of a high overexpression of wt p53 as well as a mutant p53 conversion into wt-like conformation and activity [5, 81] are worthy efforts in finding methods to treat cancers with the accumulated mutant p53.

## MATERIALS AND METHODS

### Cell lines and transfection

H1299 cells (ATCC) were grown in DMEM + 10% FBS (Sigma or Gibco) and transfected by Lipofectamine 2000 (Invitrogen) - 3 μl of the reagent per 3 μg total plasmid DNA for a 12-well plate well at approx. 80% cell confluence (and proportionally for other vessel sizes, including Nunc 8-well LabTek chambers). H358 cells (ATCC) were grown in RPMI + 10% FBS (Gibco) and transfected 2x by Lipofectamine 2000 (Invitrogen) - 6 μl of the reagent per 5 μg total plasmid DNA for a 6-well plate well at approx. 80% cell confluence and for the second time 24h later. MEF cells (*TP53* -/-, *MDM2* -/-) were a kind gift of prof. U. Hibner (Institut de Génétique Moléculaire de Montpellier, France), were grown in DMEM + 10% FBS (Sigma) and were transfected by Effectene (Qiagen) according to the manufacturer's manual. MCF7, SkBr3 and MDA-MB-468 (ATCC) cells were grown in DMEM + 10% FBS (Sigma).

### Plasmids, cloning and mutagenesis

In FRET vectors the wt p53 coding sequence was inserted to the pECFP-N1 and pEYFP-N1 (C-terminal p53 tagging, stop codon was omitted) or pECFP-C1 and pEYFP-C1 (N-terminal p53 tagging) vector backbones (Clontech). For untagged p53 variants – wt p53 or del90 p53 (generated by PCR) was inserted to the pECFP-N1 vector backbone, with removed sequence encoding ECFP. pLenti vectors with N-terminally FLAG-tagged p53 variants were a kind gift of dr M. Olszewski (International Institute of Molecular and Cell Biology in Warsaw, Poland). For the experiments shown in Supplementary Figure 2D the pLenti vectors were co-trasfected by Lipofectamine 2000 to H1299 with vectors encoding untagged p53s for transient overexpression of the p53 variants. For all types of vectors all the point mutant p53 variants were generated by a site-directed mutagenesis.

### FRET sensitized emission measurements and spectra correction/normalization

Cells from a standard size 12-well plate well (Corning) were trypsinized 24h post transfection, washed 2x with PBS and resuspended in PBS: 0.3 ml for measurements in quartz cuvettes by RF-5301 Shimadzu spectrofluorimeter (Figure 1) or 0.1 ml for measurements in v-bottom black 384-well plates (Grainer) by Tecan M1000 spectrofluorimeter (Figure 2). The measurement parameters were: for CFP – excitation wavelength 425 nm, emission wavelength range 450-550 nm, for YFP - excitation wavelength 485 nm, emission wavelength range 495-550 nm. Correction for crosstalk (cross-excitation and a spectral bleed-through form the CFP excitation to the YFP emission) was performed as shown and explained step-by-step in Supplementary Figure 1A. Spectra were normalized in the 0-1 range using the equation:

$$CFP^{norm\;\lambda n}\;=\frac{CFP^{corr\;\lambda n}-\;CFP^{corr\;\lambda 450}}{MAX\;(CFP^{corr\;\lambda 450}:\;CFP^{corr\;\lambda 550})-\;CFP^{corr\;\lambda 450}}$$

where:

*CFP^norm λn^* is the normalized CFP emission value at the wavelength of n nm in the range of the measured CFP emission spectrum (450-550 nm),

*CFP^corr λn^* is the crosstalk-corrected CFP emission value at the wavelength of n nm in the range of the measured CFP emission spectrum (450-550 nm)

*CFP^corr^* ^λ 450^ is the crosstalk-corrected CFP emission value at the wavelength of 450 nm

*MAX*(*CFP^corr^* ^λ 450^ : *CFP^corr^* ^λ 550^) is the maximum crosstalk-corrected CFP emission value in the wavelength range of the measured CFP emission spectrum (450-550 nm)

“FRET signal” values were obtained by subtraction of the crosstalk-corrected and normalized CFP spectra values at the wavelength of λ=527 nm (YFP maximum emission) of the average p53-CFP sample values from the measured sample values.

Theoretical FRET saturation values were calculated based on previous study [30] using the equation:

$$FRET^{signal}\;=\frac{\left[{\left(nYFP+nCFP\right)}^{n}-\;nYFP^{n}-nCFP^{n}\right]}{\left[{\left(nYFP+nCFP\right)}^{n}-\;nYFP^{n}-nCFP^{n}+\;n*nCFP^{n}\right]}$$

where:

*FRET^signal^* is the calculated FRET signal value

*nYFP* is the number of YFP acceptor molecules

*nCFP* is the number of CFP donor molecules

*n* is the number of monomers in an oligomer under consideration

The theoretical FRET saturation value points were calculated for used molar ratios between transfected vectors (in the range of 1:5 to 5:1 acceptor:donor). Then these values were normalized to maximum predicted values of measured FRET saturation values and non-linear regression one-phase association curves were fitted in Graph Pad Prism 6.01 to both – measured and theoretical model value points to obtain the graph in Figure 1D.

### Confocal microscopy and FRET acceptor photobleaching measurements

Leica TCS SP 2 microscope and 63x oil immersion objective was used to obtain cell photos and measure FRET efficiency using the acceptor photobleaching protocol. All photos/measurements were done in living cells, grown and transfected in 8-well Lab-Tek chambers (Nunc), on a microscope support table heated to 37°C. 24h post transfection, directly before the measurements, the medium was exchanged to DMEM + 10%FBS with 25 mM HEPES pH 7.5 to maintain the near-physiological pH outside of the CO_2_ incubator during the 2-4h microscopy sessions. Optionally Hoechst 33342 dye was added to the medium to stain nuclei. 405 nm UV laser was used for CFP excitation (collected emission range 445-502 nm) and (if used) Hoechst 33342 excitation (collected emission range 423-475 nm). 514 nm laser was used for YFP excitation (collected emission range 523-581 nm). Acceptor photobleaching was performed using the Leica Software wizard, YFP signal was set for bleaching to 30% of the original intensity, the FRET efficiency was calculated automatically by the software in the set ROIs (see Supplementary Figure 3 for additional comments on ROI and cell selection) by the standard equation:

$$FRET_{eff}=\frac{D_{post}-D_{pre}}{D_{post}}\text{ for all }D_{post}>D_{pre}$$

Where *FRET_eff_* is the FRET efficiency, *D_post_* is the measured donor (CFP) intensity post-bleaching and *D_pre_* is the donor (CFP) intensity pre-bleaching.

### Western blot and p53 stability measurement

The cells were lysed on ice post FRET measurements (after spinning down from the PBS suspension, Figure 2) or after scraping for the well (Figures 4–5) in the buffer containing 150 mM NaCl, 1% Triton X-100, 50 mM HEPES pH 8.0 and protease inhibitor cocktail (Sigma). Protein concentrations were measured in the lysates by Bradford assay (Biorad), Laemmli sample buffer was added, lysates were incubated at 95°C for 10 min and equal amounts of the total protein were loaded on the SDS-PAGE gel. The western blot protein detection was performed using the following antibodies at indicated concentrations and incubation times. Anti-p53: DO-1 (sc-126, Santa Cruz, 1:4000, 1h), DO-12 (a kind gift of prof. B. Vojtesek, Masaryk Memorial Cancer Institute, Brno, Czech Republic; 1:2000, ON), CM1 (a kind gift of prof. B.Vojtesek, 1:1000, ON). Anti-GAPDH (MAB374, Millipore, 1:2000, ON). Detection was performed with anti-mouse/rabbit secondary antibodies fused with HRP (Biorad). Band densitometry was performed using ImageJ software. p53 stability and half-life determination was done as described previously [45].

### Co-immunoprecipitation

The experiments were performed by lysing cells from 6-well plate wells, 48h post transfection, in the Co-IP buffer (150 mM NaCl, 50 mM Tris-HCl pH 8, 1mM EDTA, 0.5% NP40, 10% glycerol) with protease inhibitor cocktail (Sigma) for 15 min on ice, with additional mechanical lysis using fine-needle syringes. Lysates were pre-cleared by 1h rotation with agarose-protein G beads (washed with the Co-IP buffer), centrifuged for 20 min at 13000 g at 4°C (to clear the beads and the cell debris), input samples were collected and the main lysates were incubated with rotation overnight at 4°C with the 0.5 μg anti-GFP antibody (sc-9996, Santa Cruz) or anti-FLAG antibody (sc-166355, Santa Cruz). The agarose-protein G beads (washed with the Co-IP buffer) were added and the lysates were rotated for additional 1h. The beads were spun down, washed 3x with the co-IP buffer, resuspended in the Laemmli sample buffer, incubated at 95°C for 5 min, and the whole samples were loaded to the SDS-PAGE gel for the western blot. Inputs were blotted in parallel.

### RNA extraction and qPCR

RNA was extracted from H1299 or H358 cells scraped from the standard 6-well plate well 48h post transfection (in the case of H358 cells - post second transfection) with TRI reagent (Ambion). Half of the cells from a well were taken for protein extraction, see western blot. cDNA was produced using QuantiTect Reverse Transcription Kit (Qiagen). qPCR was carried out using Sensitive RT PCR Mix (A&A Biotechnology). *CDKN1A*, *MDM2, BCC3*, *BAX,* *PSMA2*, *PSMC1, RRM1, TK1, CCNA2 and ACTB* (house-keeping normalizer) gene mRNA-specific primer sequences are listed in the table attached in the Supplementary Data.

### Wound-healing (scratch) assay

30h post transfection of the H1299 or 24h post second transfection of the H358 cells in the standard 6-well plate, scratches were done using a 10 μl pipette tip, the medium was changed to fresh and the scratches were photographed under a Leica DM IL microscope (0h time-point; the cells were at 90-100% confluence at this time). 18h (H1299) or 42h (H358) later the scratches were photographed again. In the case of H1299 the cells from the same wells were then used to extract proteins for western blots and RNA for qPCR at the total time of 48h post transfection. The area of the cell-free scratch was measured on the photos by an ImageJ plugin MRI Wound Healing Tool (http://dev.mri.cnrs.fr/projects/imagej-macros/wiki/Wound_Healing_Tool). The cell free area at 18/42h was subtracted from the cell-free area at 0h of the same well to obtain the “wound area covered by cells post 18h/42h” values in Figures 4–5 and Supplementary Figure 5.

## SUPPLEMENTARY MATERIALS FIGURES AND TABLES



## Abbreviations

wt: wild-type
FRET: Forster Resonance Energy Transfer
CFP: Cyan Fluorescent Protein
YFP: Yellow Fluorescent Protein
GFP: Green Fluorescent Protein
GOF: gain-of-function
LOH: loss of heterozigosity

## References

[1] Kandoth C, McLellan MD, Vandin F, Ye K, Niu B, Lu C, Xie M, Zhang Q, McMichael JF, Wyczalkowski MA, Leiserson MD, Miller CA, Welch JS (2013). Mutational landscape and significance across 12 major cancer types. Nature.

[2] Tan H, Bao J, Zhou X (2015). Genome-wide mutational spectra analysis reveals significant cancer-specific heterogeneity. Sci Rep.

[3] Walerych D, Napoli M, Collavin L, Del Sal G (2012). The rebel angel: mutant p53 as the driving oncogene in breast cancer. Carcinogenesis.

[4] Walerych D, Lisek K, Del Sal G (2015). Mutant p53: one, no one, and one hundred thousand. Front Oncol.

[5] Mantovani F, Walerych D, Sal GD (2017). Targeting mutant p53 in cancer: a long road to precision therapy. FEBS J.

[6] Friedman PN, Chen X, Bargonetti J, Prives C (1993). The p53 protein is an unusually shaped tetramer that binds directly to DNA. Proc Natl Acad Sci U S A.

[7] McLure KG, Lee PW (1998). How p53 binds DNA as a tetramer. EMBO J.

[8] Kawaguchi T, Kato S, Otsuka K, Watanabe G, Kumabe T, Tominaga T, Yoshimoto T, Ishioka C (2005). The relationship among p53 oligomer formation, structure and transcriptional activity using a comprehensive missense mutation library. Oncogene.

[9] Aramayo R, Sherman MB, Brownless K, Lurz R, Okorokov AL, Orlova EV (2011). Quaternary structure of the specific p53-DNA complex reveals the mechanism of p53 mutant dominance. Nucleic Acids Res.

[10] Shaulian E, Zauberman A, Ginsberg D, Oren M (1992). Identification of a minimal transforming domain of p53: negative dominance through abrogation of sequence-specific DNA binding. Mol Cell Biol.

[11] Nicholls CD, McLure KG, Shields MA, Lee PW (2002). Biogenesis of p53 involves cotranslational dimerization of monomers and posttranslational dimerization of dimers. Implications on the dominant negative effect. J Biol Chem.

[12] Willis A, Jung EJ, Wakefield T, Chen X (2004). Mutant p53 exerts a dominant negative effect by preventing wild-type p53 from binding to the promoter of its target genes. Oncogene.

[13] Sabapathy K (2015). The contrived mutant p53 oncogene - beyond loss of functions. Front Oncol.

[14] Chan WM, Siu WY, Lau A, Poon RY (2004). How many mutant p53 molecules are needed to inactivate a tetramer?. Mol Cell Biol.

[15] Natan E, Hirschberg D, Morgner N, Robinson CV, Fersht AR (2009). Ultraslow oligomerization equilibria of p53 and its implications. Proc Natl Acad Sci U S A.

[16] Rajagopalan S, Huang F, Fersht AR (2011). Single-Molecule characterization of oligomerization kinetics and equilibria of the tumor suppressor p53. Nucleic Acids Res.

[17] Gaglia G, Guan Y, Shah JV, Lahav G (2013). Activation and control of p53 tetramerization in individual living cells. Proc Natl Acad Sci U S A.

[18] Gaglia G, Lahav G (2014). Constant rate of p53 tetramerization in response to DNA damage controls the p53 response. Mol Syst Biol.

[19] Deb D, Scian M, Roth KE, Li W, Keiger J, Chakraborti AS, Deb SP, Deb S (2002). Hetero-oligomerization does not compromise ‘gain of function’ of tumor-derived p53 mutants. Oncogene.

[20] Lang GA, Iwakuma T, Suh YA, Liu G, Rao VA, Parant JM, Valentin-Vega YA, Terzian T, Caldwell LC, Strong LC, El-Naggar AK, Lozano G (2004). Gain of function of a p53 hot spot mutation in a mouse model of Li-Fraumeni syndrome. Cell.

[21] Olive KP, Tuveson DA, Ruhe ZC, Yin B, Willis NA, Bronson RT, Crowley D, Jacks T (2004). Mutant p53 gain of function in two mouse models of Li-Fraumeni syndrome. Cell.

[22] Wang Y, Suh YA, Fuller MY, Jackson JG, Xiong S, Terzian T, Quintas-Cardama A, Bankson JA, El-Naggar AK, Lozano G (2011). Restoring expression of wild-type p53 suppresses tumor growth but does not cause tumor regression in mice with a p53 missense mutation. J Clin Invest.

[23] Turrell FK, Kerr EM, Gao M, Thorpe H, Doherty GJ, Cridge J, Shorthouse D, Speed A, Samarajiwa S, Hall BA, Griffiths M, Martins CP (2017). Lung tumors with distinct p53 mutations respond similarly to p53 targeted therapy but exhibit genotype-specific statin sensitivity. Genes Dev.

[24] Miyawaki A, Tsien RY (2000). Monitoring protein conformations and interactions by fluorescence resonance energy transfer between mutants of green fluorescent protein. Methods Enzymol.

[25] Vogel SS, Thaler C, Koushik SV (2006). Fanciful FRET. Sci STKE.

[26] Raicu V (2007). Efficiency of resonance energy transfer in homo-oligomeric complexes of proteins. J Biol Phys.

[27] Drinovec L, Kubale V, Nohr Larsen J, Vrecl M (2012). Mathematical models for quantitative assessment of bioluminescence resonance energy transfer: application to seven transmembrane receptors oligomerization. Front Endocrinol (Lausanne).

[28] Amiri H, Schultz G, Schaefer M (2003). FRET-based analysis of TRPC subunit stoichiometry. Cell Calcium.

[29] Alvarez-Curto E, Ward RJ, Pediani JD, Milligan G (2010). Ligand regulation of the quaternary organization of cell surface M3 muscarinic acetylcholine receptors analyzed by fluorescence resonance energy transfer (FRET) imaging and homogeneous time-resolved FRET. J Biol Chem.

[30] Fung JJ, Deupi X, Pardo L, Yao XJ, Velez-Ruiz GA, Devree BT, Sunahara RK, Kobilka BK (2009). Ligand-regulated oligomerization of beta(2)-adrenoceptors in a model lipid bilayer. EMBO J.

[31] Albizu L, Cottet M, Kralikova M, Stoev S, Seyer R, Brabet I, Roux T, Bazin H, Bourrier E, Lamarque L, Breton C, Rives ML, Newman A (2010). Time-resolved FRET between GPCR ligands reveals oligomers in native tissues. Nat Chem Biol.

[32] Huang F, Rajagopalan S, Settanni G, Marsh RJ, Armoogum DA, Nicolaou N, Bain AJ, Lerner E, Haas E, Ying L, Fersht AR (2009). Multiple conformations of full-length p53 detected with single-molecule fluorescence resonance energy transfer. Proc Natl Acad Sci U S A.

[33] Dudek JM, Horton RA (2010). TR-FRET biochemical assays for detecting posttranslational modifications of p53. J Biomol Screen.

[34] Chung HS, Meng F, Kim JY, McHale K, Gopich IV, Louis JM (2017). Oligomerization of the tetramerization domain of p53 probed by two- and three-color single-molecule FRET. Proc Natl Acad Sci U S A.

[35] Yamauchi M, Suzuki K, Kodama S, Watanabe M (2005). Abnormal stability of wild-type p53 protein in a human lung carcinoma cell line. Biochem Biophys Res Commun.

[36] Tracz-Gaszewska Z, Klimczak M, Biecek P, Herok M, Kosinski M, Olszewski MB, Czerwinska P, Wiech M, Wiznerowicz M, Zylicz A, Zylicz M, Wawrzynow B (2017). Molecular chaperones in the acquisition of cancer cell chemoresistance with mutated TP53 and MDM2 up-regulation. Oncotarget.

[37] Davison TS, Yin P, Nie E, Kay C, Arrowsmith CH (1998). Characterization of the oligomerization defects of two p53 mutants found in families with Li-Fraumeni and Li-Fraumeni-like syndrome. Oncogene.

[38] Mercier JF, Salahpour A, Angers S, Breit A, Bouvier M (2002). Quantitative assessment of beta 1- and beta 2-adrenergic receptor homo- and heterodimerization by bioluminescence resonance energy transfer. J Biol Chem.

[39] Bouaoun L, Sonkin D, Ardin M, Hollstein M, Byrnes G, Zavadil J, Olivier M (2016). TP53 variations in human cancers: new lessons from the IARC TP53 database and genomics data. Hum Mutat.

[40] Cho Y, Gorina S, Jeffrey PD, Pavletich NP (1994). Crystal structure of a p53 tumor suppressor-DNA complex: understanding tumorigenic mutations. Science.

[41] Bullock AN, Henckel J, Fersht AR (2000). Quantitative analysis of residual folding and DNA binding in mutant p53 core domain: definition of mutant states for rescue in cancer therapy. Oncogene.

[42] Bullock AN, Fersht AR (2001). Rescuing the function of mutant p53. Nat Rev Cancer.

[43] O’Keefe K, Li H, Zhang Y (2003). Nucleocytoplasmic shuttling of p53 is essential for MDM2-mediated cytoplasmic degradation but not ubiquitination. Mol Cell Biol.

[44] Kruiswijk F, Labuschagne CF, Vousden KH (2015). p53 in survival, death and metabolic health: a lifeguard with a licence to kill. Nat Rev Mol Cell Biol.

[45] Walerych D, Lisek K, Sommaggio R, Piazza S, Ciani Y, Dalla E, Rajkowska K, Gaweda-Walerych K, Ingallina E, Tonelli C, Morelli MJ, Amato A, Eterno V (2016). Proteasome machinery is instrumental in a common gain-of-function program of the p53 missense mutants in cancer. Nat Cell Biol.

[46] Lisek K, Campaner E, Ciani Y, Walerych D, Del Sal G (2018). Mutant p53 tunes the Nrf2-dependent antioxidant response to support survival of cancer cells. Oncotarget.

[47] Kollareddy M, Dimitrova E, Vallabhaneni KC, Chan A, Le T, Chauhan KM, Carrero ZI, Ramakrishnan G, Watabe K, Haupt Y, Haupt S, Pochampally R, Boss GR (2015). Regulation of nucleotide metabolism by mutant p53 contributes to its gain-of-function activities. Nat Commun.

[48] Liu K, Ling S, Lin WC (2011). TopBP1 mediates mutant p53 gain of function through NF-Y and p63/p73. Mol Cell Biol.

[49] Muller PA, Caswell PT, Doyle B, Iwanicki MP, Tan EH, Karim S, Lukashchuk N, Gillespie DA, Ludwig RL, Gosselin P, Cromer A, Brugge JS, Sansom OJ (2009). Mutant p53 drives invasion by promoting integrin recycling. Cell.

[50] Yeudall WA, Wrighton KH, Deb S (2013). Mutant p53 in cell adhesion and motility. Methods Mol Biol.

[51] Bado I, Nikolos F, Rajapaksa G, Gustafsson JA, Thomas C (2016). ERbeta decreases the invasiveness of triple-negative breast cancer cells by regulating mutant p53 oncogenic function. Oncotarget.

[52] Mishra AK, Mavlyutov T, Singh DR, Biener G, Yang J, Oliver JA, Ruoho A, Raicu V (2015). The sigma-1 receptors are present in monomeric and oligomeric forms in living cells in the presence and absence of ligands. Biochem J.

[53] Mishra AK, Gragg M, Stoneman MR, Biener G, Oliver JA, Miszta P, Filipek S, Raicu V, Park PS (2016). Quaternary structures of opsin in live cells revealed by FRET spectrometry. Biochem J.

[54] Stoneman MR, Paprocki JD, Biener G, Yokoi K, Shevade A, Kuchin S, Raicu V (2017). Quaternary structure of the yeast pheromone receptor Ste2 in living cells. Biochim Biophys Acta.

[55] Xu J, Reumers J, Couceiro JR, De Smet F, Gallardo R, Rudyak S, Cornelis A, Rozenski J, Zwolinska A, Marine JC, Lambrechts D, Suh YA, Rousseau F (2011). Gain of function of mutant p53 by coaggregation with multiple tumor suppressors. Nat Chem Biol.

[56] Wiech M, Olszewski MB, Tracz-Gaszewska Z, Wawrzynow B, Zylicz M, Zylicz A (2012). Molecular mechanism of mutant p53 stabilization: the role of HSP70 and MDM2. PLoS One.

[57] Saha T, Guha D, Manna A, Panda AK, Bhat J, Chatterjee S, Sa G (2016). G-actin guides p53 nuclear transport: potential contribution of monomeric actin in altered localization of mutant p53. Sci Rep.

[58] Kato S, Han SY, Liu W, Otsuka K, Shibata H, Kanamaru R, Ishioka C (2003). Understanding the function-structure and function-mutation relationships of p53 tumor suppressor protein by high-resolution missense mutation analysis. Proc Natl Acad Sci U S A.

[59] Coffill CR, Muller PA, Oh HK, Neo SP, Hogue KA, Cheok CF, Vousden KH, Lane DP, Blackstock WP, Gunaratne J (2012). Mutant p53 interactome identifies nardilysin as a p53R273H-specific binding partner that promotes invasion. EMBO Rep.

[60] Zhao Y, Zhang C, Yue X, Li X, Liu J, Yu H, Belyi VA, Yang Q, Feng Z, Hu W (2015). Pontin, a new mutant p53-binding protein, promotes gain-of-function of mutant p53. Cell Death Differ.

[61] Polotskaia A, Xiao G, Reynoso K, Martin C, Qiu WG, Hendrickson RC, Bargonetti J (2015). Proteome-wide analysis of mutant p53 targets in breast cancer identifies new levels of gain-of-function that influence PARP, PCNA, and MCM4. Proc Natl Acad Sci U S A.

[62] Rivlin N, Katz S, Doody M, Sheffer M, Horesh S, Molchadsky A, Koifman G, Shetzer Y, Goldfinger N, Rotter V, Geiger T (2014). Rescue of embryonic stem cells from cellular transformation by proteomic stabilization of mutant p53 and conversion into WT conformation. Proc Natl Acad Sci U S A.

[63] van Dieck J, Fernandez-Fernandez MR, Veprintsev DB, Fersht AR (2009). Modulation of the oligomerization state of p53 by differential binding of proteins of the S100 family to p53 monomers and tetramers. J Biol Chem.

[64] Lui K, Sheikh MS, Huang Y (2015). Regulation of p53 oligomerization by Ras superfamily protein RBEL1A. Genes Cancer.

[65] Muller P, Chan JM, Simoncik O, Fojta M, Lane DP, Hupp T, Vojtesek B (2018). Evidence for allosteric effects on p53 oligomerization induced by phosphorylation. Protein Sci.

[66] Varley JM, Thorncroft M, McGown G, Appleby J, Kelsey AM, Tricker KJ, Evans DG, Birch JM (1997). A detailed study of loss of heterozygosity on chromosome 17 in tumours from Li-Fraumeni patients carrying a mutation to the TP53 gene. Oncogene.

[67] Schlegelberger B, Kreipe H, Lehmann U, Steinemann D, Ripperger T, Gohring G, Thomay K, Rump A, Di Donato N, Suttorp M (2015). A child with Li-Fraumeni syndrome: modes to inactivate the second allele of TP53 in three different malignancies. Pediatr Blood Cancer.

[68] Nakayama M, Sakai E, Echizen K, Yamada Y, Oshima H, Han TS, Ohki R, Fujii S, Ochiai A, Robine S, Voon DC, Tanaka T, Taketo MM (2017). Intestinal cancer progression by mutant p53 through the acquisition of invasiveness associated with complex glandular formation. Oncogene.

[69] Alexandrova EM, Mirza SA, Xu S, Schulz-Heddergott R, Marchenko ND, Moll UM (2017). p53 loss-of-heterozygosity is a necessary prerequisite for mutant p53 stabilization and gain-of-function *in vivo*. Cell Death Dis.

[70] Shetzer Y, Kagan S, Koifman G, Sarig R, Kogan-Sakin I, Charni M, Kaufman T, Zapatka M, Molchadsky A, Rivlin N, Dinowitz N, Levin S, Landan G (2014). The onset of p53 loss of heterozygosity is differentially induced in various stem cell types and may involve the loss of either allele. Cell Death Differ.

[71] Parikh N, Hilsenbeck S, Creighton CJ, Dayaram T, Shuck R, Shinbrot E, Xi L, Gibbs RA, Wheeler DA, Donehower LA (2014). Effects of TP53 mutational status on gene expression patterns across 10 human cancer types. J Pathol.

[72] Monti P, Perfumo C, Bisio A, Ciribilli Y, Menichini P, Russo D, Umbach DM, Resnick MA, Inga A, Fronza G (2011). Dominant-negative features of mutant TP53 in germline carriers have limited impact on cancer outcomes. Mol Cancer Res.

[73] Stoczynska-Fidelus E, Szybka M, Piaskowski S, Bienkowski M, Hulas-Bigoszewska K, Banaszczyk M, Zawlik I, Jesionek-Kupnicka D, Kordek R, Liberski PP, Rieske P (2011). Limited importance of the dominant-negative effect of TP53 missense mutations. BMC Cancer.

[74] Navone NM, Troncoso P, Pisters LL, Goodrow TL, Palmer JL, Nichols WW, von Eschenbach AC, Conti CJ (1993). p53 protein accumulation and gene mutation in the progression of human prostate carcinoma. J Natl Cancer Inst.

[75] Top B, Mooi WJ, Klaver SG, Boerrigter L, Wisman P, Elbers HR, Visser S, Rodenhuis S (1995). Comparative analysis of p53 gene mutations and protein accumulation in human non-small-cell lung cancer. Int J Cancer.

[76] Alsner J, Jensen V, Kyndi M, Offersen BV, Vu P, Borresen-Dale AL, Overgaard J (2008). A comparison between p53 accumulation determined by immunohistochemistry and TP53 mutations as prognostic variables in tumours from breast cancer patients. Acta Oncol.

[77] López I, P Oliveira L, Tucci P, Alvarez-Valín F, A Coudry R, Marín M (2012). Different mutation profiles associated to P53 accumulation in colorectal cancer. Gene.

[78] Bouchalova P, Nenutil R, Muller P, Hrstka R, Appleyard MV, Murray K, Jordan LB, Purdie CA, Quinlan P, Thompson AM, Vojtesek B, Coates PJ (2014). Mutant p53 accumulation in human breast cancer is not an intrinsic property or dependent on structural or functional disruption but is regulated by exogenous stress and receptor status. J Pathol.

[79] Liu J, Li W, Deng M, Liu D, Ma Q, Feng X (2016). Immunohistochemical Determination of p53 Protein Overexpression for predicting p53 gene mutations in hepatocellular carcinoma: a meta-analysis. PLoS One.

[80] Terzian T, Suh YA, Iwakuma T, Post SM, Neumann M, Lang GA, Van Pelt CS, Lozano G (2008). The inherent instability of mutant p53 is alleviated by Mdm2 or p16INK4a loss. Genes Dev.

[81] Bykov VJ, Eriksson SE, Bianchi J, Wiman KG (2018). Targeting mutant p53 for efficient cancer therapy. Nat Rev Cancer.

