# Supplementary material for: Wild-type p53 oligomerizes more efficiently than p53 hot-spot mutants and overcomes mutant p53 gain-of-function via a “dominant-positive” mechanism
Source: Oncotarget. 2018 Aug 10;9(62):32063–80. doi: 10.18632/oncotarget.25944 (PMC6112834; doi:10.18632/oncotarget.25944)
Supplement: Supplementary file 1 [file oncotarget-09-32063-s001.pdf]

Wild-type p53 oligomerizes more efficiently than p53 hot-spot mutants and overcomes mutant p53 gain-of-function via a "dominant-positive" mechanism

SUPPLEMENTARY MATERIALS

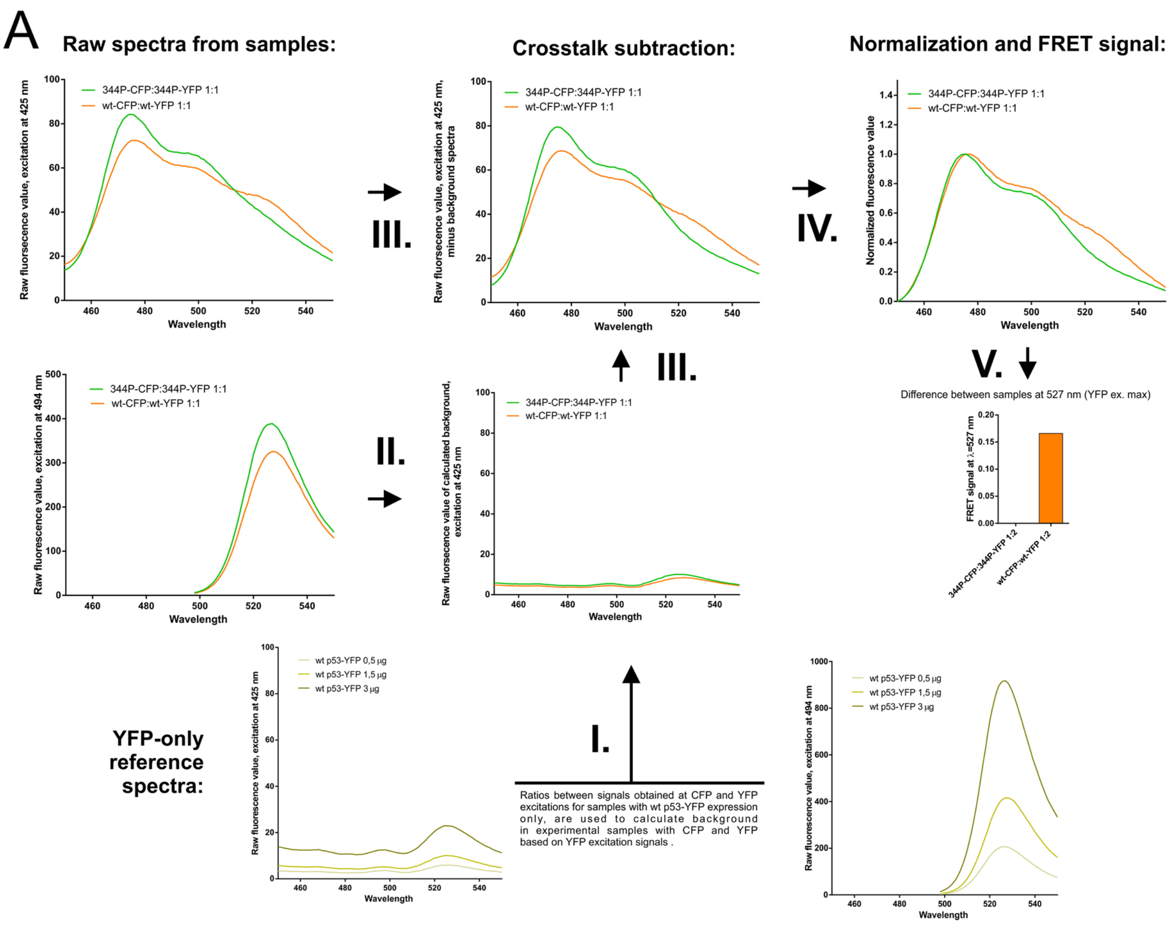

Supplementary Figure 1: (A) The procedure of CFP spectra correction and normalization in the spectrofluorimetric sensitized emission experiments is shown schematically using examples of wt-CFP:wt-YFP (tetrameric p53, orange spectra) and p53 344P-CFP:344P-YFP (monomeric p53, green spectra) constructs transfected to H1299 cells at 1:1 molar ratio. (I.) The YFP spectra were collected from a series of p53-YFP overexpression experiments (0.5, 1.5 and 3 µg of transfected wt-YFP plasmid per 3cm well of cells are shown), without CFP, to cover the range of typical YFP intensities obtained in the CFP:YFP experiments (YFP excitation at λ=485 nm). For the same cells spectra were collected using the CFP excitation wavelength (λ=410 nm). The ratio of both spectra was calculated in the range of the measured YFP emission spectrum (495-550 mn). The ratio values were found to be very similar at each λ value for the shown 0.5-3 µg range of the transfected p53-Y, indicating nearly linear CFP to YFP spectrum cross-talk. Hence, the averages of the ratios were used for further calculations (e.g. at λ=527 the average ratio of CFP-range background spectrum to YFP spectrum was 0.026, with SD=0.0020; at λ=498 aver. ratio=0.997, SD= 0.087382; at λ=550 aver. ratio=0.035, SD= 0.002693). The remaining CFP-range background spectrum (450-497 nm) was calculated by using the constant ratio (0.997) at the 498 nm. The result was the CFP-range background spectrum, which could be calculated for any CFP:YFP experimental setup by (II.) measuring the YFP spectrum excited at λ=485 nm. (III.) Such spectra calculated separately for each experiment were subtracted from the raw CFP-range spectra in the experimental samples. (IV.) These background- and crosstalk-corrected CFP spectra were then normalized in the 0-1 range (see Materials and Methods) and (V.) FRET signal values were calculated at λ=527 nm.

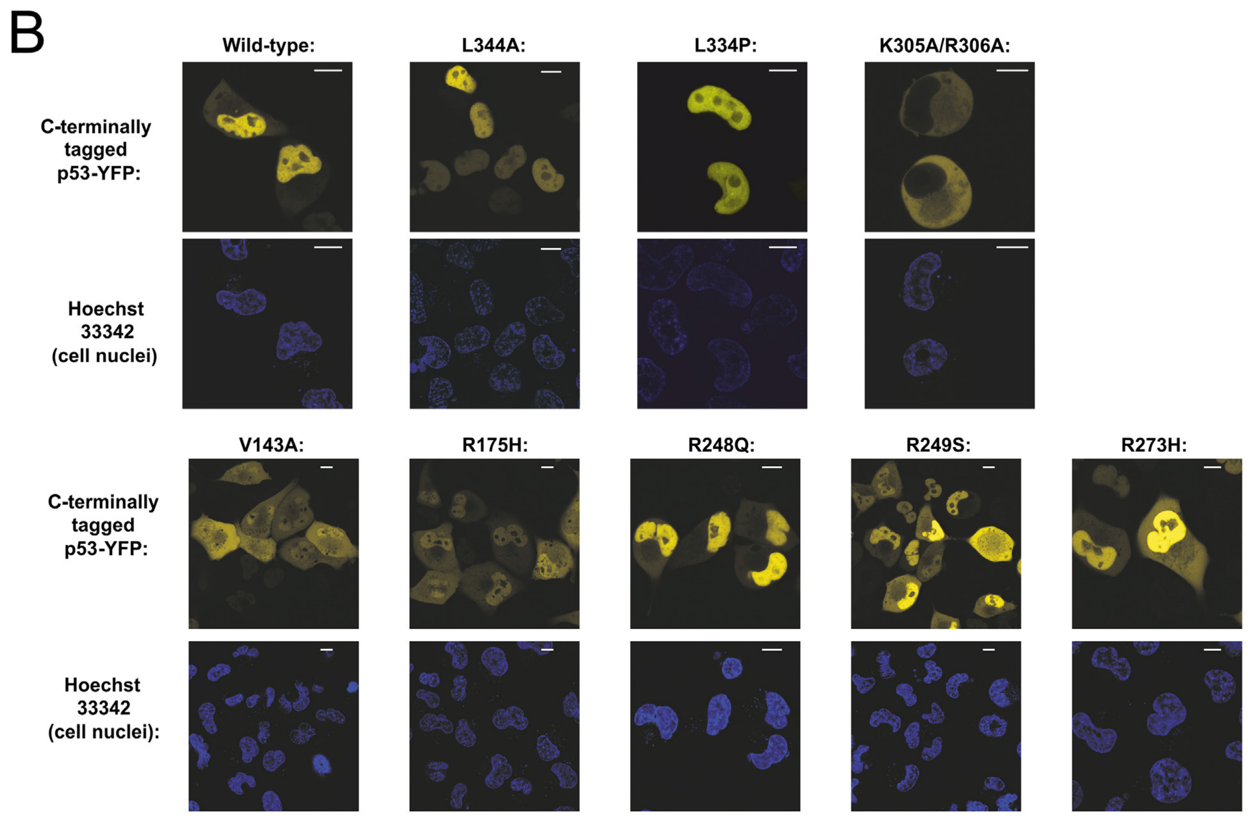

Supplementary Figure 1: (Continued) **(B)** Sub-cellular localization of the used p53-YFP constructs is shown in live, transfected H1299 cells grown in Lab-Tek chambers, using confocal microscopy. The localization of the p53-CFP constructs was the same (see Figure 3 or not-shown). Yellow – YFP signal, blue – Hoechst dye added to medium. Bar size = 10 μM.

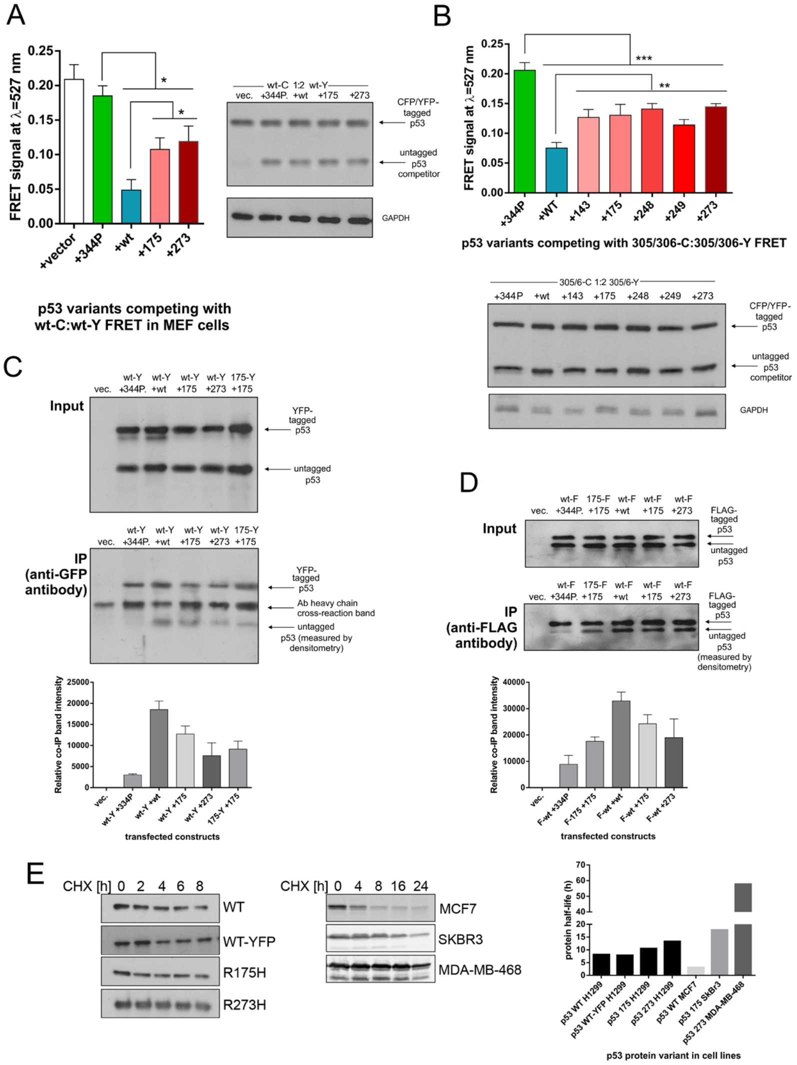

Supplementary Figure 2: (A) The FRET competition assay in MEF *TP53* -/- *MDM2* -/- cells with p53 wt:wt (0.5 µg wt-C : 1 µg wt-Y) oligomers using indicated series of untagged constructs (1.5 µg co-transfected with FRET constructs). Averages of 2 biological replicates are shown in the graph with SD, statistical significance was calculated with one-way ANOVA, Bonferroni post-test, * p-value<0.05. Right panel: western blot analysis for p53 (DO-1 antibody) and GAPDH (housekeeping protein) of a total protein lysate from a representative replicate of the FRET measurements. **(B)** The FRET competition assay in H1299 cells with predominantly cytoplasmic p53 305/306 variant (0.5 µg 305/306-C : 1 µg 305/306-Y) oligomers using indicated series of untagged constructs (1.5 µg co-transfected with FRET constructs). Averages of 2 biological replicates are shown with SD, statistical significance was calculated with one-way ANOVA, Bonferroni post-test, *** p-value<0.001, ** p-value<0.01. Lower panel: western blot analysis for p53 (DO-1 antibody) and GAPDH (housekeeping protein) of a total protein lysate from a representative replicate of the FRET measurements shown in the graph and a purified, untagged p53 (positive control). **(C)** Co-immunoprecipitaion of indicated C-terminally YFP-tagged p53 variants with untagged p53 variants overexpressed in H1299 cells, using anti-GFP antibody (mouse origin). The input is blotted in the upper panel and the co-IP - in the lower panel. Detection was done using rabbit anti-p53 polyclonal CM1 antibody (the IP antibody heavy chain was detected in the cross-reaction post co-IP). The bar graph shows densitometry result averages of two experiments with SD of the relative intensity (with subtracted lane 1 background) of the immunoprecipitated untagged p53 variant band. Wt-Y does not co-precipitate with the monomeric variant p53 L344P while it co-precipitates with p53 variants wt, R175H and R273H. R175H-Y and R175H co-precipitation is also detectable. **(D)** Co-immunoprecipitaion of indicated N-terminally FLAG-tagged p53 variants with untagged p53 variants overexpressed in H1299 cells, using anti-FLAG antibody (mouse origin). The input is blotted in the upper panel and the co-IP - in the lower panel. Detection was done using rabbit anti-p53 polyclonal CM1 antibody. The bar graph shows densitometry result averages of two experiments with SD of the relative intensity (with subtracted lane 1 background) of the immunoprecipitated untagged p53 variant band. F-wt p53 co-precipitates more strongly with p53 wt than R175H and R273H. F-175H and 175H co-precipitation is also detectable. **(E)** Western blot panels show p53 protein bands (detected by DO-1 antibody) at indicated time post treatment with cycloheximide (CHX) in indicated cell lines: overexpressed ectopically in H1299 cells (left panel) or endogenous p53 variants - wt in MCF7 cells, R175H in SkBr3 cells and R273H in MDA-MB-468 cells. In the bar graph calculated protein half-lives are shown – indicating that in H1299 cells the wt p53 stability is increased and more similar to mutant variants, compared to endogenous p53 variants in tested breast cancer cells.

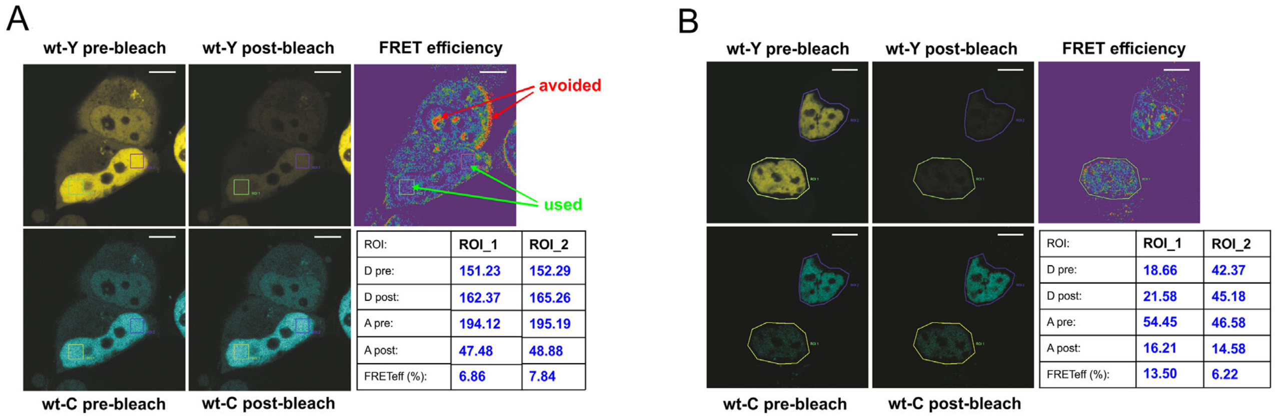

Supplementary Figure 3: **(A)** An example how regions of local high FRET efficiency and cells with mislocalized proteins were avoided during the experiments. The photos show results of the acceptor photobleaching FRET efficiency measurement in live H1299 cells growing in a Lab-Tek chamber, transfected with vectors overexpressing wt-C and wt-Y (vector molar proportion 1:2). Pre- and post-bleach photos are shown for the CFP channel (donor; blue) and the YFP channel (acceptor; yellow) – the whole two central cells were photobleached. FRET efficiency color map shows small patches of high local FRET efficiency (red; marked with red arrows) which were avoided thanks to positioning of the used ROIs 1 and 2 in the regions of intensive and uniform fluorophore signals (green arrows). Additionally – the upper cell was avoided due to unusual, low presence of wt-C/Y p53 in the cell's nucleus (compare with the lower cell and Figure 3). The results table shows the FRET efficiency calculated for the chosen ROI areas. Bar size - 10 µm. **(B)** An example how high difference in YFP:CFP ratio was avoided in the experiments. The photos show results of the acceptor photobleaching FRET efficiency measurement in live H1299 cells growing in a Lab-Tek chamber, transfected with vectors overexpressing wt-C and wt-Y (vector molar proportion 1:2). Pre- and post-bleach photos are shown for the CFP channel (donor; blue) and the YFP channel (acceptor; yellow) – the whole two central cells were photobleached. FRET efficiency color map and the result table shows high FRET efficiency in the lower cell, with unusually high YFP:CFP ratio due to the low CFP signal intensity (ROI 1). Such cells, with non-typical signal ratios or distributions, found in low numbers, were avoided during the experiments. Bar size - 10 µm.

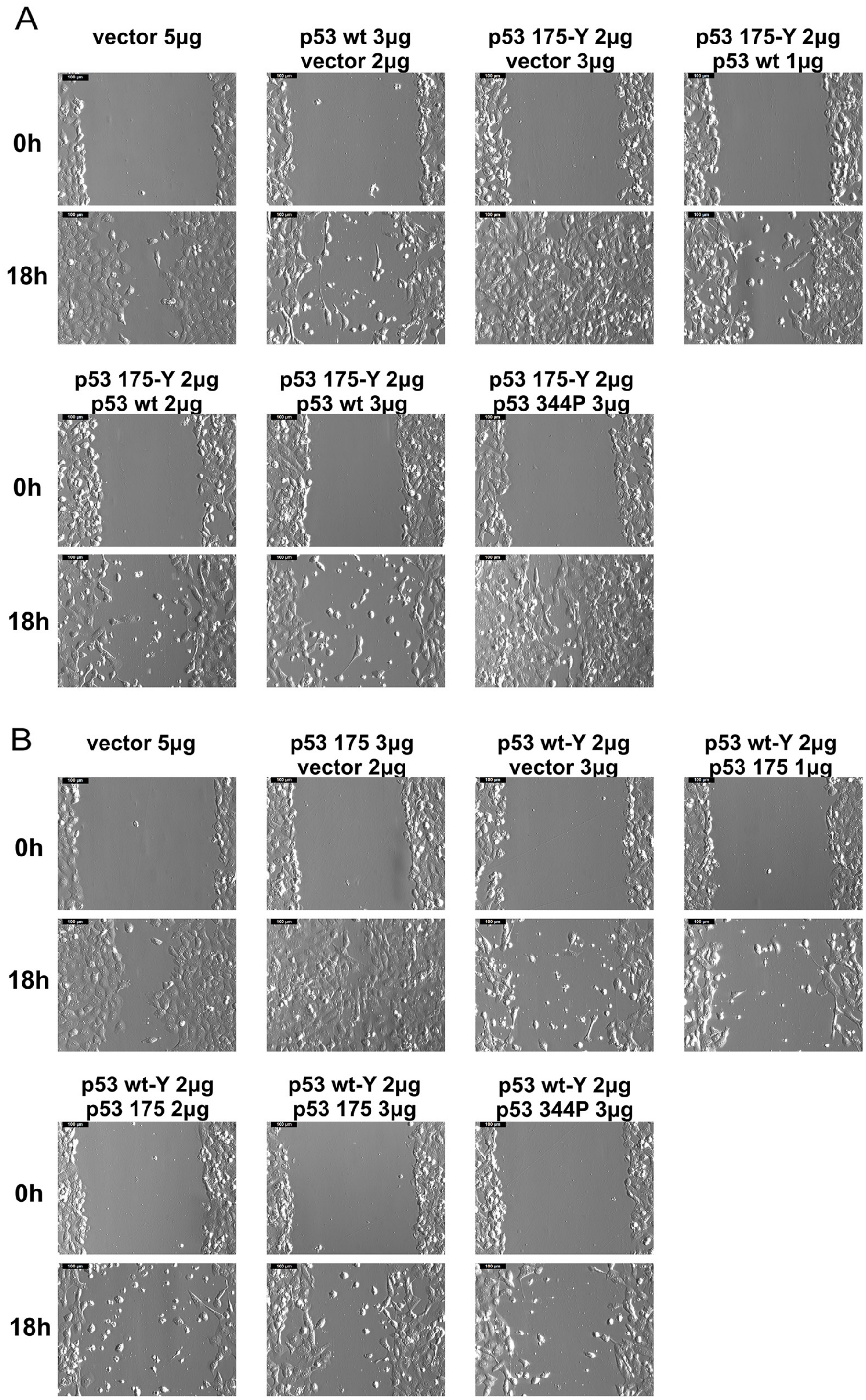

Supplementary Figure 4: (A) Representative photos of the wound-healing (scratch) assay performed in H1299 cells used to produce cell migration bar graph in Figure 4A. 30h post transfection with indicated vectors scratches were done and photographed (0h) and after the next 18h the scratches were photographed again (18h). Cell-free areas were measured for both time-points by the Wound-healing plug-in for ImageJ (Materials and Methods) and the covered area used in the bar graph was calculated by subtraction of the cell-free area at 18h from the cell-free area at 0h. The bar size is 100 µm. **(B)** Representative photos of the wound-healing (scratch) assay performed in H1299 cells used to produce cell migration bar graph in Figure 4B. The assay was done as described in (A). The bar size is 100 µm.

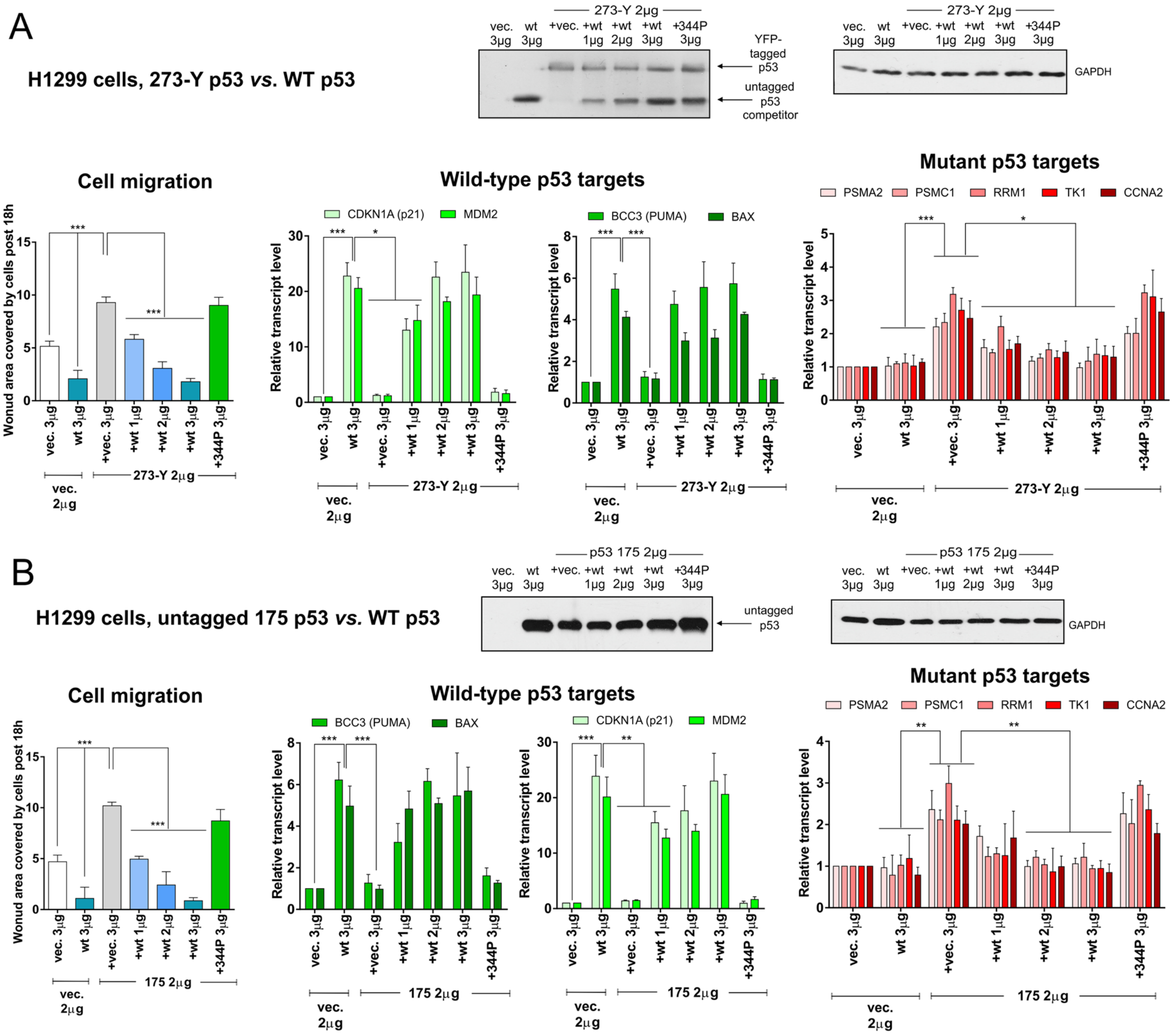

Supplementary Figure 5: **(A)** H1299 cells were transfected with indicated amounts of empty vector (vec.) and/or vectors encoding p53 protein variants – untagged wt or 344P with/without 273-YFP mutant. The western blot shows p53 (DO-1 antibody) and GAPDH (housekeeping control) levels in the representative competition titration experiment. The cell migration graph shows average results with SD of area covered by cells post 18h of the wound-healing (scratch) assay in 3 biological replicates of the wt p53 titration vs p53 273-Y mutant variant. Expression bar graphs show normalized averages with SD of the mRNA levels for wt p53 target genes (strongly induced: *CDKN1A* and *MDM2;* moderately induced: *BCC3* and *BAX*) and mutant p53 target genes (*PSMA2, PSMC1, RRM1, TK1, CCNA2*) in 3 biological replicates of the wt p53 titration vs p53 273-Y mutant variant. Statistical significance was calculated with one-way ANOVA, Bonferroni post-test, *** p-value<0.001, * p-value<0.05. The result extends the result shown in Figure 4A by demonstrating that significant inhibition of the R273H mutant p53 GOF effects (gene expression and cell migration induction) by wt p53 is present at sub-equimolar amount of wt *vs.* mutant p53. **(B)** H1299 cells were transfected with indicated amounts of empty vector (vec.) and/or vectors encoding p53 protein variants – untagged wt, 344P with/without untagged R175H mutant. The western blot shows p53 (DO-1 antibody) and GAPDH (housekeeping control) levels in the representative competition titration experiment. The cell migration graph shows average results with SD of area covered by cells post 18h of the wound-healing (scratch) assay in 3 biological replicates of the wt p53 titration vs p53 175 mutant variant. Expression bar graphs show normalized averages with SD of the mRNA levels for wt p53 target genes (strongly induced: *CDKN1A* and *MDM2;* moderately induced: *BCC3* and *BAX*) and mutant p53 target genes (*PSMA2, PSMC1, TK1*) in 3 biological replicates of the wt p53 titration vs p53 175 mutant variant. Statistical significance was calculated with one-way ANOVA, Bonferroni post-test, *** p-value<0.001, ** p-value<0.01. The result confirms the result shown in Figure 4A by demonstrating that significant inhibition of the untagged R175H mutant p53 GOF effects (gene expression and cell migration induction) by untagged wt p53 is present at equimolar amount of wt *vs.* mutant p53.

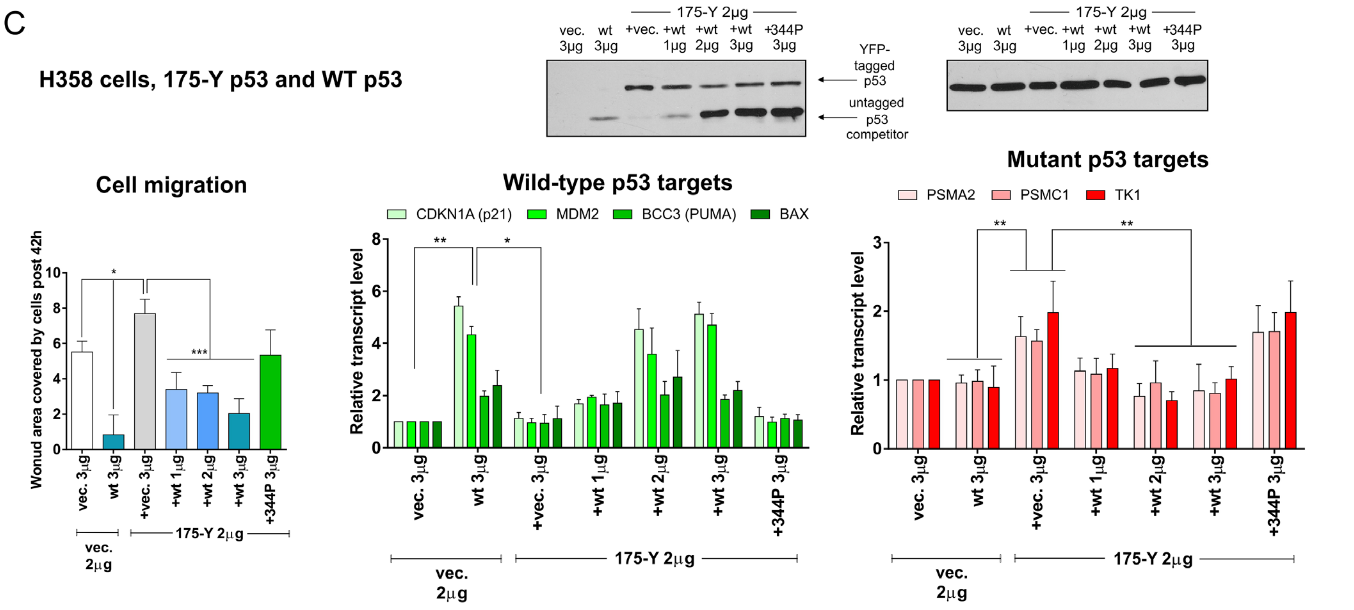

Supplementary Figure 5: (Continued) (C) H358 cells were transfected with indicated amounts of empty vector (vec.) and/or vectors encoding p53 protein variants – untagged wt or 344P with/without 175-YFP mutant. The western blot shows p53 (DO-1 antibody) and GAPDH (housekeeping control) levels in the representative competition titration experiment. The cell migration graph shows average results with SD of area covered by cells post 42h of the wound-healing (scratch) assay in 3 biological replicates of the wt p53 titration vs p53 175-Y mutant variant. Expression bar graphs show normalized averages with SD of the mRNA levels for wt p53 target genes (strongly induced: *CDKN1A* and *MDM2;* moderately induced: *BCC3* and *BAX*) and mutant p53 target genes (*PSMA2, PSMC1, TK1*) in 3 biological replicates of the wt p53 titration vs p53 175-Y mutant variant, 48h post second transfection. Statistical significance was calculated with one-way ANOVA, Bonferroni post-test, *** p-value<0.001, ** p-value<0.01, * p-value<0.05. The result extends the result shown in Figure 4A by demonstrating that significant inhibition of the R175H mutant p53 GOF effects (gene expression and cell migration induction) by wt p53 is present at equimolar amount of wt *vs.* mutant p53 in H358 cells background.

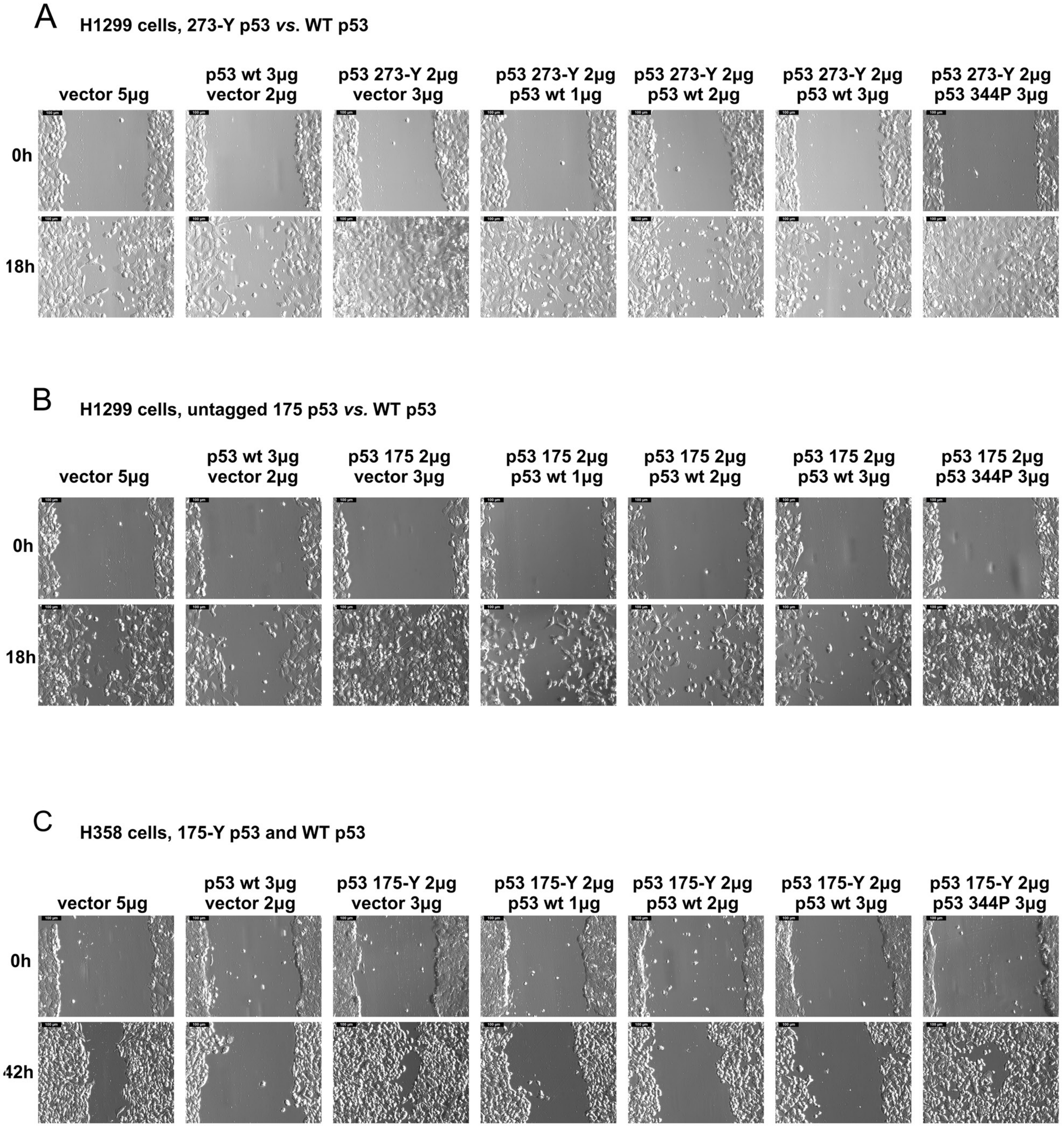

Supplementary Figure 6: **(A)** Representative photos of the wound-healing (scratch) assay performed in H1299 cells used to produce cell migration bar graph in Supplementary Figure 5A. 30h after transfection with indicated vectors scratches were done and photographed (0h) and after the next 18h the scratches were photographed again (18h). Cell-free areas were measured for both time-points by the Wound-healing plug-in for ImageJ (Materials and Methods) and the covered area used in the bar graph was calculated by subtraction of the cell-free area at 18h from the cell-free area at 0h. **(B)** Representative photos of the wound-healing (scratch) assay performed in H1299 cells used to produce cell migration bar graph in Supplementary Figure 5B. The assay was done as described in (A). **(C)** Representative photos of the wound-healing (scratch) assay performed in H358 cells used to produce cell migration bar graph in Supplementary Figure 5C. 24h after a second transfection with indicated vectors scratches were done and photographed (0h) and after the next 42h the scratches were photographed again (42h). Cell-free areas were measured for both time-points by the Wound-healing plug-in for ImageJ (Materials and Methods) and the covered area used in the bar graph was calculated by subtraction of the cell-free area at 18h from the cell-free area at 0h. The bar size is 100 μm.

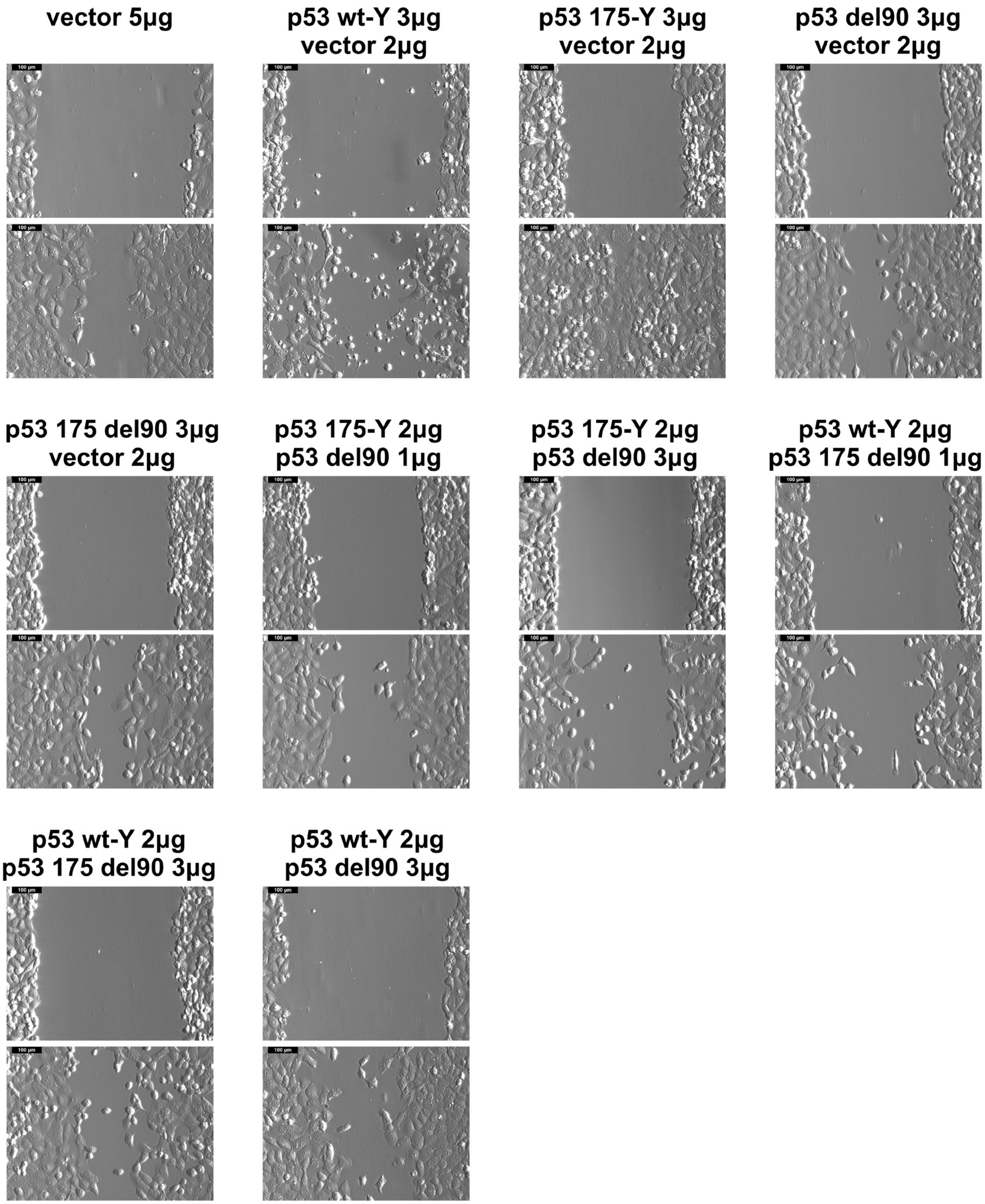

Supplementary Figure 7: Representative photos of the wound-healing (scratch) assay performed in H1299 cells used to produce cell migration bar graph in Figure 5. 30h post transfection with indicated vectors scratches were done and photographed (0h) and after the next 18h the scratches were photographed again (18h). Cell-free areas were measured for both time-points by the Wound-healing plug-in for ImageJ (Materials and Methods) and the covered area used in the bar graph was calculated by subtraction of the cell-free area at 18h from the cell-free area at 0h. The bar size is 100 µm.

Supplemenatry Table 1: qPCR primer pairs

Target gene name	Forward primer	Reverse primer
CDKN1A	GGACCTGTCACTGTCTTGTA	GGCTTCCTCTTGGAGAAGAT
BCC3	GAGCGGCGGAGACAAGAG	TGGGTAAGGGCAGGAGTC
MDM2	CTTCTCTGTGAAAGAGCACAGG	CCGATGATTCCTGCTGATTG
BAX	ATGCGTCCACCAAGAAGC	GGCGGCAATCATCCTCTG
PSMA2	GTGCTTTGGCTCTTCGGGTA	GCTTTAATTCCCACGGACGG
PSMC1	TGGAGCTTCCTCTCACCCAT	TGGCTGAGGTTTGGTTTGCT
RRM1	AGAAGATTGCAAAGTATGGT	GTAAGGTTCAATGGACTCAT
TK1	CAATGAGCTGCATTAACCT	GTGTCTTTGGCATACTTGAT
CCNA2	AAGCAGAAAAAGAAGCTCAGAAGA	GACATGTCCATAGTATGTGGTGACT
ACTB	CGCCGCCAGCTCACCATG	CACGATGGAGGGGAAGACGG
